# Synchronized nutrition fertilizer improves soil microbial diversity and sugarcane yield through agrochemical and enzymatic regulation

**DOI:** 10.3389/fpls.2025.1694590

**Published:** 2025-10-02

**Authors:** Xiuxiu Qi, Jiaquan Jiang, Di Yang, Qingbo Li, Chengxiang Gao, Lidan Zhang, Shaolong Sun, Xiaolin Fan

**Affiliations:** ^1^ College of Natural Resources and Environment, South China Agricultural University, Guangzhou, China; ^2^ Guangdong Engineering Technology Research Center of Low Carbon Agricultural Green Inputs, South China Agricultural University, Guangzhou, China; ^3^ School of Agricultural Engineering, Guangxi Vocational & Technical College, Nanning, China

**Keywords:** synchronized nutrition fertilizer, conventional chemical fertilizer, sugarcane yield, soil acidification, microbial diversity

## Abstract

**Introduction:**

The new type of synchronized nutrition fertilizers (SNFert) has been popularized and applied in agro-production in northern China. However, the impacts of the SNFert on soil microbial diversity and sugarcane growth have not yet been thoroughly investigated.

**Methods:**

To clarify the influences of SNFert on soil properties, sugarcane yield, soil microbial diversity and their interactions, two years of field experiments with four treatments, including CK, non fertilizer; CF, conventional chemical fertilizer; SNF1, 15% less fertilizer applied than CF; SNF2, 25% less than CF, were conducted.

**Results:**

Results showed that yield, °Brix in the SNFert, even with 15% and 25% less fertilizer input, were no significant differences with those of CF. Specifically, SNF1 increased sugarcane yield and tillers by 6.84% and 18.63%, raised pH and urease by 0.40 units and 2.29 times, reduced Nmin and EC by 81.69% and 77.69% with 15% lower fertilizer input, respectively. In 2020, soil microbial diversity was enriched by SNFert through regulating microbial communities and functions. As a new kind of chemical fertilizer, SNF1 overcame the shortcomings of CF in reducing the soil microbial diversity, that was, there were no statistically differences in microbial alpha diversity indices between SNF1 and CK. The abundance of beneficial functional taxa *Chloroflexi*, *Proteobacteria*, *Acidobacteriota* in bacteria and *Mortierellomycota* in fungi of SNF1 increased by 12.46%, 15.64%, 47.63% and 164.00%, respectively. The soil carbon and nitrogen cycle was driven by those taxa. The plant-microbe nutritional exchanges were then improved. In mechanism, SNFert not only supplemented nutrients, but also enriched soil microbial diversity by increasing soil pH and decreasing soil salinity and mineral nutrient residues, thereby improving soil properties and increasing sugarcane yield in 2020.

**Discussion:**

In conclusion, replacing CF with SNFert in sugarcane cultivation is an effective fertilization measure to reduce the dosage of CF, optimize soil agrochemical properties, and increase soil microbial diversity.

## 1 Introduction

China is a major sugar consumer, and sugarcane is one of the main sources of sugar. The proportion of sugarcane in the sugar cultivation area and output accounted for approximately 90% annually (Qi et al., 2022; Li et al., 2024). Approximately 1.1362 million hectares of sugarcane have been mainly cultivated in non-irrigated hilly areas in China (FAO, 2021). Production conditions are poor for the crop growth (Wang et al., 2022). A large amount of nutrients and organic matter had been moved out from the soil with each harvest (Luo et al., 2022), while return of straw (leaves, and stalks) was limited (Tabriz et al., 2021). Thus, sugarcane production mainly depends on extensive use of conventional or chemical fertilizers (CF) (Singh et al., 2019). Soil fertility, fertilizer use efficiency, soil microbial diversity have been decreased by long-term monoculture and overuse of the CF (Takeda et al., 2021; Wu et al., 2021; Muhammad et al., 2022). Pang et al. (2021) and Yang et al. (2021) reported that widespread soil degradation and yield stagnation had existed in the sugarcane production regions. Therefore, how to improve soil properties and increase soil microbial diversity through chemical fertilizer innovation and fertilization strategy is urgent to achieve sustainable and high-yield production of sugarcane in China.

Crop productivity correlated closely with soil microbial diversity (Fan et al., 2021). Increasing soil microbial diversity would enhance soil nutrient availability and plant productivity (Delgado-Baquerizo et al., 2020; Sekulić et al., 2023). Soil microorganisms play a crucial role in maintaining soil function, quality and ecosystem (Ji et al., 2022; Li et al., 2023) by rapidly responding to changes in the soil environment (Tayyab et al., 2018). It was known that prolonged CF application in continuous sugarcane cultivation had resulted in soil acidification and soil fertility decline. Both reduction of sugarcane quality and yield, and soil degradation would be the results of a decrease in microbial diversity and beneficial microbial taxa (Pang et al., 2021; Khan et al., 2023; Zhang et al., 2024). Soil pH was one of the key factors that impacted on soil bacterial communities (Tan et al., 2020). Niu et al. (2021) reported that an increase in soil pH could raise soil microbial biomass and abundance of beneficial microbial taxa, thereby increasing sugarcane productivity. Application of slow-release fertilizer (SRF) and controlled-release fertilizer (CRF) could also improve soil physical and chemical properties, thereby improving the soil microbial diversity (Yuan et al., 2023). As a result, soil fertility could be maintained and crop yields could be increased (Niu et al., 2022). It is to say that the key to increasing sugarcane yield sustainably in China will depend on synchronized nutrition fertilizer (SNFert) application, which supplies nutrients synchronously with sugarcane demand and fosters soil microbial growth.

SNFert gained recognition as a nutrient efficient fertilizer after launch of the action plan for zero-growth of chemical fertilizer during the “13^th^ Five-Year Plan” in China (Liu et al., 2019; Chen et al., 2020). SNFert is a kind of fertilizer derived from bio-oil coated controlled release NPK compound and CF. The characteristic of SNFert is synchronized nutrient supply with crop demand. Specifically, SNFer can synchronously provide nitrogen, phosphorus and potassium with a proper ratio and quantity consistent with the demand of each stage in the three major growth phases of vegetative organ growth, vegetative and reproductive organ co-growth, and reproductive organ growth (Fan, 2012; Jiang et al., 2022). In a wheat–maize planting system in the north China plain, SNFert significantly increased the winter wheat grain yields by 4.29%–14.69%, and the summer maize grain yields by 12.67%–18.50% (Zhang et al., 2023). The TN (1.13 g·kg^-1^–1.64 g·kg^-1^), TP (0.37 g·kg^-1^–0.43 g·kg^-1^), AN (49.45 mg·kg^-1^–68.84 mg·kg^-1^), AP (7.26 mg·kg^-1^–10.09 mg·kg^-1^), and AK contents were significantly enhanced under SNFert treatments (Zhang et al., 2025). For N loss in SNFert treatments, N_2_O emission and N leaching were reduced by 0.04 kg·ha^-1^–0.27 kg·ha^-1^ and 1.90 kg·ha^-1^–9.40 kg·ha^-1^ (Wang et al., 2019). Therefore, the SNFert would be beneficial to soil physical and chemical properties, and increase crop yield and fertilizer use efficiency. Although SNFert has been popularized in northern China, the application of sugarcane-specific SNFert remains to be further studied on its effects on soil microbial diversity and soil agrochemical properties. It is worth noting that Husted et al. (2024) proposed that agriculture research, not only nanotechnology-based, but also including CRF or SRF, should be designed a reasonable nutrient-application regimes, and should be hypothesis-driven and focus on providing mechanistic insights rather than just observing effects of a given treatment of soil and plants. The research idea of this paper is exactly the same as that of Husted et al. (2024). The authors assume that the application of the SNFert will be a driving force to raise sugarcane yield and quality. It may be achieved through the following two aspects. SNFert can increase yield through its special nutrient supply patterns that nutrient supply is able to meet the sugarcane demand. That is to say, the nutrient supply by SNFert is basically in synchronized with the demand of the crops. Concurrently, SNFert can enhance soil microbial diversity by improving the soil conditions for soil microbial growth. Therefore, this study mainly focuses on how sugarcane yield responds to the interaction between SNFert and soil microbial communities in the rhizosphere through two years field experiments. The objectives were to elucidate the mechanisms how SNFert influenced sugarcane yield, soil properties and microbial diversity. The results of the study would also be expected to provide theoretical guidance for rational fertilization and high yield and quality through optimizing the sugarcane fertilization mode with SNFert.

## 2 Materials and methods

### 2.1 Experimental site and materials

Field trial was carried out in China sugarcane experiment station in Longan County, Guangxi Province in 2020 and 2021. The soil is lateritic red soil. The soil pH, organic matter, total N, P and K, available N, P and K were 4.61, 27.4 g·kg^-1^, 1.4 g·kg^-1^, 0.5 g·kg^-1^, 1.0 g·kg^-1^, 93 mg·kg^-1^, 24.6 mg·kg^-1^ and 269.2 mg·kg^-1^, respectively. The annual temperature and average rainfall were 22.4°C and 1300.9 mm, 22.8°C and 1037.7 mm in 2020 and 2021, respectively.

The sugarcane variety was the local main cultivar Guitang 40. The fertilizers in the test were conventional chemical fertilizers (CF) and SNFert (SNF), respectively.

### 2.2 Experimental design

A two-factor comparative design was implemented in the experiment over two consecutive years. The first factor, SNFert, comprised two levels which are 15% less fertilizer applied than CF (labeled as SNF1) and 25% less than CF (labeled as SNF2). The CF was the second factor and recognized as conventional fertilizer control. Non fertilizer was set as absolute control (CK). There were four treatments in total, each treatment included three replicates being three plots (10 m × 6 m per plot). 5 rows of sugarcane were planted in each plot, and the row spacing was 120 cm. Each row was planted with 54 seedling-stem with double-bud on each stem. The planting furrow was 50 cm in width and 30 cm in depth. The base fertilizers were applied before the placement and then covered with 5 cm of soil.

N: P_2_O_5_: K_2_O ratio of the fertilizer was 1: 0.77: 1.1 (**Table 1**), and the total amount of NPK fertilizer application was 2508.3 kg·ha^-1^. Base and topdressing fertilizers accounted for 60% and 40%, respectively. The base fertilizers of CF contained compound fertilizer (15-15-15), potassium chloride (0-0-60) and calcium magnesium phosphate fertilizer (0-18-0). The ratios among the three fertilizers was 28%, 23%, and 49%, respectively. The topdressing was urea and potassium chloride. The base fertilizers of SNF1 and SNF2 were specialized SNFert for sugarcane, which were developed independently by the author’s research center. The base fertilizer consisted of PU coated controlled-release fertilizer (CRF) (14.5-14.5-14.5), water soluable compound fertilizer (15-15-15), potassium chloride (0-0-60) and calcium magnesium phosphate fertilizer (0-18-0). The ratio of the four fertilizers was 4%, 31%, 6% and 59%, respectively. The coating material of the PU coated CRF were 80% castor + 20% soybean oil as hydroxyl compounds and isocyanate (Wei et al., 2021). The nutrient composition (N, P_2_O_5_, K_2_O) of all fertilizers applied is shown in **Table 1**. The nutrient inputs were adjusted for total nutrient equivalence. The topdressing was composed of coated urea and coated potassium chloride. All treatments maintained an identical N: P_2_O_5_: K_2_O ratio during the growth period. The total amount of NPK inputs in SNF1 and SNF2 treatments was 1848.89 kg·ha^-1^ and 1631.11 kg·ha^-1^, respectively.

**Table 1:** Nutrient allocation and amount of fertilizers.

Fertilizer type	Nutrition ratio			Dosage (kg·ha^-1^)		
	N	P_2_O_5_	K_2_O	N	P_2_O_5_	K_2_O
CF	1	0.77	1.1	552	426	602
SNF1				407	314	444
SNF2				359	277	457

CK, CF, SNF1 and SNF2 stand for non fertilizer, conventional fertilizer, 15% less than CF and 20% less than CF.

The experiment in 2020 was started on March 11^th^ with seedling transplanting. The topdressing was on June 10^th^ with replenished soil at the base of the sugarcane. Harvest date was on December 4^th^. The seedling transplanting, topdressing and harvest dates were on March 8^th^, June 10^th^ and December 8^th^ in 2021. The growth period of sugarcane in two years was 263 days. The other cultivation practices were conducted consistent with local farmers’ habits.

### 2.3 Sample collection and determination

Plant samples were collected in 2020 and 2021, respectively. The number of basic seedlings, total seedlings and stalks was counted at the seedling, tillering and maturation stages, respectively. The sucrose content (°Brix) and actual yield of sugarcane were measured at harvest. The representative sugarcane stalks were selected as samples in each plot by checkerboard method at harvest. Roots, stems, dead leaves and green leaves were then separated and collected after oven-drying at 75 °C to constant weight, and weighed.

Soil samples were also collected at the sugarcane harvest in 2020 and 2021, respectively. Rhizosphere soil sampling was taken when collecting plant samples as follows. A soil profile was excavated to 20 cm depth using a steel shovel, positioned 10 cm from the sugarcane stem. Then gently pull out the root system with the shovel. The soil not adhering to the root surface (non-rhizosphere soil) would naturally fall off. The root-adhering soil after shaking the roots in a clean plastic bag would be collected as rhizosphere soil. The rhizosphere soil of 10 plants was collected together and fully mixed, and immediately sieved (2 mm). Soil samples of each treatment were taken from mixed samples and placed in an ice box immediately. The samples were stored in a refrigerator at -80 °C after returning to the laboratory. According to the literature, in a long-term stable ecosystem, altering nutrient input and soil properties would trigger an immediate response from the microbial community, and at this time, the changes in the microbial community were the most significant (Han et al., 2023; Su et al., 2025). Once the structure and function of the soil microbial community were formed, they usually remained relatively stable for 1 to 5 years (Gschwend et al., 2021; Fox et al., 2022). Meanwhile, according to the research reports by Han et al. (2023) and Su et al. (2025), the results of soil microbial diversity in the first year after applying SNFert could more directly reflect the impact of SNFert on soil microbial diversity. Therefore, the determination of microbial diversity (16S rRNA and ITS sequencing) in this study referred to the research report of Fox et al. (2022), that was, the soil samples were collected and measured in the first year (2020) after the application of SNFert. Concurrently, approximately 500 g of soil was collected in 2020 and 2021, respectively, and stored at 4 °C for the determination of enzyme activity. Another 500 g of soil was taken and air-dried for the determination of the other soil’s chemical properties.

### 2.4 Determination items and methods

The number of tillers was calculated as follows:


$$Tiller\;number\;(No.per\cdot ha)=N_{1}-N_{0}$$

Where N_1_ is the total number of sugarcane seedlings, N_0_ is the basic number of sugarcane seedlings.

The actual sugarcane yield was determined by harvesting all plants in each plot. The yield per hectare was calculated as follows:


$$Actual\;yield\;(t\cdot ha^{-1})=\frac{Yield\;a\;plot(kg)\times 10000}{60\times 1000}$$

The °Brix of each year was measured by Extech Portable Sucrose Brix Refractometer (Mid-State Instruments, CA, USA) at harvest.

Soil pH and EC value were determined according to Deng et al. (2022). Mineral nitrogen (Nmin) was extracted with saturated CaCl_2_, and nitrate (NO_3_^−^-N) and ammonium (NH_4_^+^-N) in the solution were determined using a continuous flow analyzer (CFA, AMD Paris, France). The available phosphorus (AP) was determined following Watanabe and Olsen (1965). Available potassium (K^+^) was determined according to Pansu (2006). Soil organic matter (SOM) was measured according to Fan et al. (2022). Exchangeable Ca^2+^ and Mg^2+^ were extracted with NH_4_OAc and the Ca^2+^ and Mg^2+^ in the leaching solution were determined by atomic absorption spectrophotometer (AAS). Soil urease activity was determined following Tayyab et al. (2018). Soil acid phosphatase activity and sucrase activity were determined according to Tayyab et al. (2021) and Zhang et al. (2019), respectively.

### 2.5 Soil DNA extraction and PCR amplification

The total DNA of soil micro-organisms was extracted by the soil DNA extraction kit (Omega Biotek, Norcross, GA, U.S.). The quality and concentration of soil DNA were tested by 1% agarose gel electrophoresis and NanoDrop 2000 spectrophotometer (Thermo Science, USA).

PCR amplification and sequencing: The purified DNA was used as a template to amplify the V3–V4 region of the bacterial 16S rRNA gene by PCR with the gene-specific primers set 338F (5’-ACTCCTACGGGAGGCAGCAG-3’)/806R (5’-GGACTACHVGGGTWTCTAAT-3’). The ITS1 region of the fungal internal transcription region (ITS) was amplified with fungus-specific primers ITS1F (CTTGGTCATTTAGAGGAAGTAA)/ITS2R (GCTGCGTTCTTCATCGATGC). The PCR (ABI GeneAmp^®^ 9700) reaction program was pre-denaturation at 95 °C for 3 min, then undergo reaction cycles process, denaturation 30s at 95°C, annealing at 55 °C for 30 s, extension at 72 °C for 45 s, bacterial 16S rRNA for 27 cycles, fungi ITS for 35 cycles, and extend 72 °C for 10 min (Bian et al., 2025). Electrophoresis was performed with 2% agarose gels to detect the specificity of amplified products. Purified amplicons were pooled in equimolar amounts and paired-end sequenced on an Illumina PE300/PE250 platform (Illumina, San Diego, USA) according to the standard protocols by Majorbio Bio-Pharm Technology Co. Ltd. (Shanghai, China).

The raw sequence was obtained by Illumina MiSeq sequencing. The original data was spliced, quality controlled and filtered to acquire effective data using the Fastp (version 1.2.11) software according to the overlap relationship. The effective data of all samples were processed by Uparse (version 7.0.1090 http://drive5.com/uparse/) software to optimize sequence redundancy calculation, remove single sequences and chimeras, and obtain the representative sequences of operational taxonomic units (OTUs) of ≥ 97% sequence similarity. The OTU representative sequences were homogenized by the RDP classifier Bayesian algorithm.

### 2.6 Data analysis

Based on the OTU data, alpha diversity indices including Sobs, Shannon, ACE and Chao1 index were calculated with Mothur v1.30.1 (Chappidi et al., 2019). Microbial diversity includes species and functional diversity. The heterogeneity among the microbial communities in different samples was determined by principal coordinate analysis (PCA). Circos plots (R: Version 3.3.1) were constructed to analyze the differences in the relative abundance of microbial composition. The linear discriminant analysis effect size (LEfSe) identified the unique microbial taxa (phylum to genus) among the different groups (Segata et al., 2011). The functions of bacterial (carbon and nitrogen cycles) were predicted using the FAPROTAX database platform (Louca et al., 2016). The bubble plots of bacterial function were created using by R package of “ggpubr” (Liu et al., 2023). The fungal functions were classified by using FUNGuild (Fungi Functional Guild) V1.0, and the differences in species abundance with the same fungal function were analyzed in different treatments (Nguyen et al., 2016). Redundancy analysis (RDA), Network, Mantel and Metabo Analyst analyses addressed the hypotheses that SNFert enhanced soil microbial diversity (community and function) by improving soil environment. RDA based on Bray-Curtis distance with Monte Carlo permutation tests (n = 999) was performed. The difference of soil bacterial and fungal community structure was evaluated by nonmetric multidimensional scaling (NMDS), based on the PERMANOVA test method (n = 999). Network analysis was performed using Mantel test in R (Deng et al., 2022). The Mantel test based on Spearman correlation and FDR correction was performed (Liu et al., 2023). Partial least squares path modeling (PLS-PM) was conducted to clarify the mechanisms that sugarcane yield responds to the interaction between soil agrochemical properties and soil microbial diversity under SNFert treatments using bootstrap validation (200 iterations). In PLS-PM, soil factors include pH, EC, Nmin, K^+^ and urease activity, microbial diversity includes bacterial and fungal alpha-diversity. The rest data were processed by Microsoft Excel. The statistical analysis was done using SPSS 19.0. A two-way analysis of variance (ANOVA) was conducted to analyze the statistical significance of the year (Y) × treatment (T) interaction for sugarcane growth and soil agrochemical properties. Error bars represent SE (n = 3). A *P* < 0.05 indicates difference at 5% level, and *P* < 0.01 indicates difference at 1% level. *P* < 0.05 was considered to be statistically significant. Figures were drawn by Origin 2021.

## 3 Results

### 3.1 Effect of SNFert on sugarcane growth

**Table 2** showed that the interaction between year and treatment was statistically significant (*P* < 0.01) for tillers and yield. The results showed that the yield and sucrose content of SNFert were similar to those of CF. Studies in two years revealed that the yield in fertilizer treatments was significantly higher than that of CK (**Table 3**). In 2020, the yield between the SNFert and CF was not significant. There was no significant difference in °Brix among the three fertilizer treatments in each year. Notably, tiller number of SNFert treatments in 2020 was significantly higher than that of CK and CF, which increased by an average of 67.96% and 19.28%, respectively. Therefore, application of the SNFert possessed great potential to increase sugarcane yield. However, the increase in economic yield of sugarcane by the SNFert depended on how to raise the stalk number eventually (**Table 3**). The possible reason for less stalk number in the SNFert treatments compared to CF was that so many tillers in the SNFert consumed too many nutrients. In addition, the total nutrients in the SNFert were less than those in CF. That was to say the nutrients in the SNFert were insufficient for so many tillers to be grown into sugarcane stalks. This conclusion was proved by the sugarcane yield in 2021. The sugarcane yield of SNF1 increased by 6.83% compared with CF. It could be concluded that as long as rational input reduction was achieved, the sugarcane yield would be significantly increased by application of the SNFert. However, optimal reduction thresholds will be a topic worthy of further study.

**Table 2:** Variance analysis of the effects of treatment (T), year (Y), and their interactions on the sugarcane yield and its constituent factors.

Sources of variation	Tiller number (No.per·ha)	Stalk number (Stalk·ha^-1^)	Total dry weight (t·ha^-1^)	Yield (t·ha^-1^)	°Brix
Year (Y)	7.3^ns^	486.7^**^	414.0^**^	84.1^**^	14.2^*^
Treatment (T)	0.2^ns^	1.9^ns^	2.1^ns^	3.2^ns^	12.8^*^
Y×T	11.5^**^	0.4^ns^	0.2^ns^	17.1^**^	0.9^ns^
Total variance	3383329630	2716703703	1049.0	8005.8	716.2

The values shown are the *F*-statistic of the analysis of variance ((ANOVA). “*” and “**” mean significant correlation at 0.05 and 0.01 levels, respectively. “ns” mean not significant.

**Table 3:** Effects of SNFert on yield and its constituent factors in the two years.

Date	Index	Treatment			
		CK	CF	SNF1	SNF2
2020	Tiller number (No.per·ha)	10889 ± 3934b	15333 ± 4178a	18189 ± 3375a	18389 ± 6140a
	Stalk number (Stalk·ha^-1^)	78556 ± 1402a	81333 ± 2186a	76222 ± 2985a	79055 ± 1402a
	Total dry weight (t·ha^-1^)	41.3 ± 0.8a	41.9 ± 1.1a	41.5 ± 0.7a	41.3 ± 0.7a
	Yield (t·ha^-1^)	103.5 ± 1.8b	109.7 ± 0.7a	110.0 ± 0.3a	108.2 ± 1.3a
	°Brix	18.8 ± 0.7a	17.7 ± 0.8b	17.4 ± 0.2b	18.0 ± 0.8b
2021	Tiller number (No.per·ha)	10989 ± 3283a	9378 ± 2845ab	9444 ± 2426ab	7878 ± 2433b
	Stalk number (Stalk·ha^-1^)	58833 ± 1084a	58389 ± 2929a	57556 ± 1982a	59167 ± 1357a
	Total dry weight (t·ha^-1^)	29.2 ± 1.6a	31.4 ± 1.5a	30.1 ± 4.4a	28.1 ± 2.5a
	Yield (t·ha^-1^)	59.4 ± 1.9c	78.1 ± 0.6b	83.4 ± 1.4a	75.0 ± 1.1b
	°Brix	18.8 ± 0.1a	18.1 ± 0.1b	18.0 ± 0.1b	18.2 ± 0.1b

CK, CF, SNF1 and SNF2 stand for non fertilizer, conventional fertilizer, 15% less than CF and 20% less than CF. The numbers are mean ± standard error (SE) (n = 15). Different lowercase letters in the same row indicate significant differences among the four treatments (*P* < 0.05).

### 3.2 Effect of SNFert on soil agrochemical properties and enzyme activity

**Figure 1:**
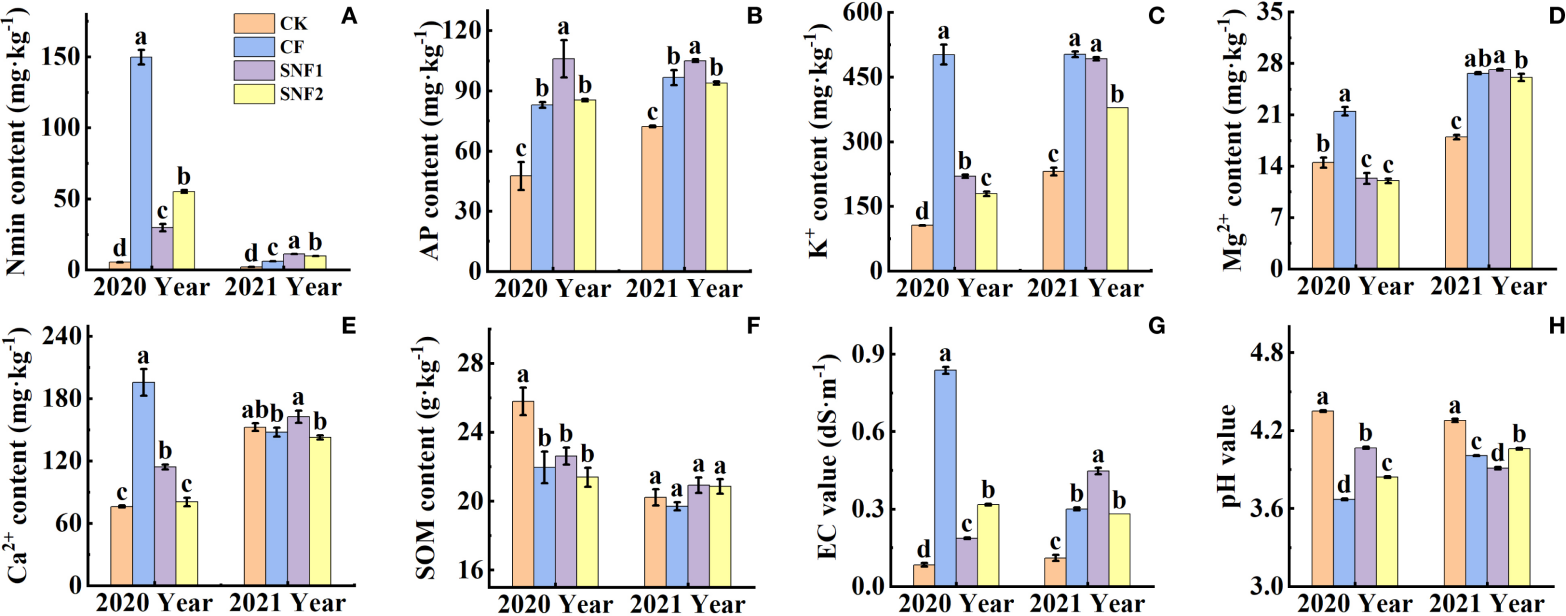
Effects of SNFert on soil Nmin **(A)**, AP **(B)**, K^+^ **(C)**, exchangeable Mg^2+^ **(D)**, and Ca^2+^ **(E)**, SOM content **(F)**, EC **(G)** and pH value **(H)** in two years. Different lowercase letters above bars in the same year indicate significant differences among treatments (*P* < 0.05, n = 3). CK, CF, SNF1 and SNF2 stand for non fertilizer, conventional fertilizer, 15% less than CF and 20% less than CF, respectively.

Soil Nmin, AP, K^+^, Ca^2+^ and Mg^2+^, EC and pH values were significantly affected by fertilization (**Figure 1**). As **Supplementary Table S1**, the interaction between year and treatment was statistically significant (*P* < 0.01) for pH, EC, Nmin, K^+^, Ca^2+^, Mg^2+^, SOM. Soil Nmin, AP, K^+^ and EC in fertilization treatments in the two years were remarkably increased, while the pH was decreased compared with CK (**Figures 1A, C, G, H**). Among fertilizer treatments, Nmin, K^+^, Ca^2+^, Mg^2+^ contents and EC of the SNFert treatments in the first year were significantly lower than those of CF (**Figures 1A, C–E, G**). The pH of SNF1 increased by 0.40 units compared with CF (**Figure 1H**). Nmin, AP and Ca^2+^ contents of SNF1 in 2021 were significantly higher than those of CF by 81.69%, 8.63% and 10.09%, respectively (**Figures 1A, B, E**). Although the EC value of SNF1 was higher than that of CF in 2021, it was still lower than that of CF in 2020 (**Figure 1G**). Conversely, the pH of SNF1 was lower than that of CF in 2021, but it was still higher than that of CF in 2020 (**Figure 1H**).

**Figure 2:**
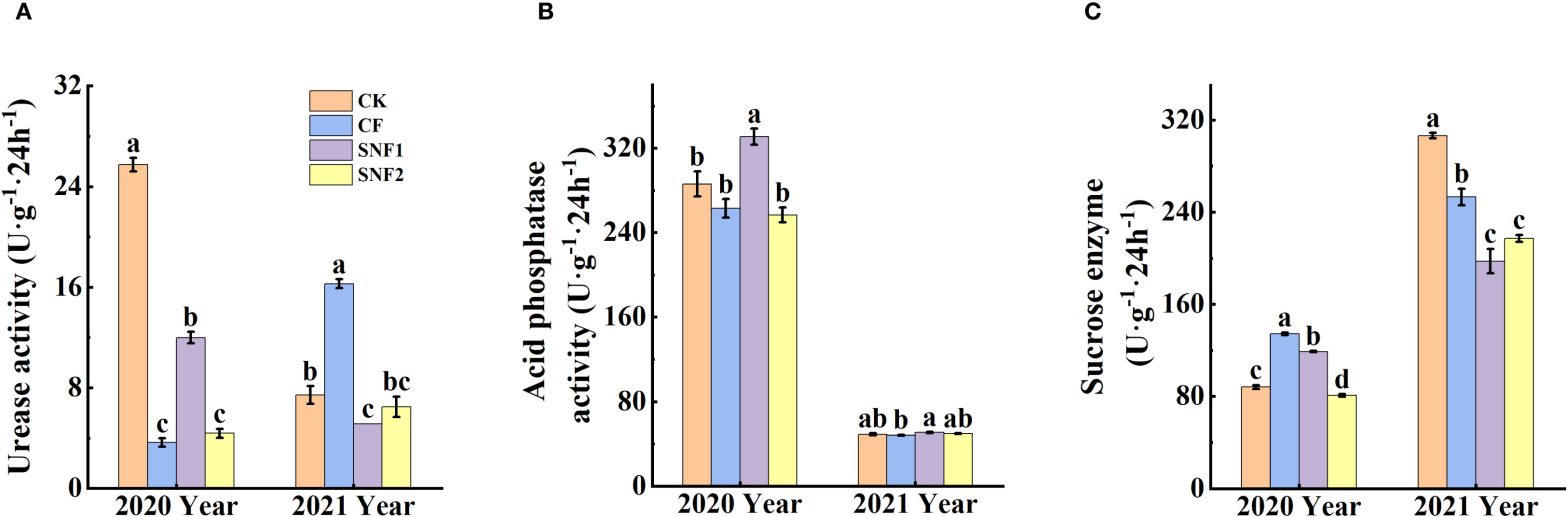
Effects of SNFert on soil urease activity **(A)**, acid phosphatase activity **(B)**, sucrase activity **(C)** in 2020 and 2021. CK, CF, SNF1 and SNF2 stand for non fertilizer, conventional fertilizer, 15% less than CF and 20% less than CF, respectively. Different lowercase letters above bars in the same year indicate significant differences among treatments (*P* < 0.05, n = 3).

**Figure 2** showed that soil urease, acid phosphatase and sucrase activity were greatly impacted by the SNFert, especially in 2020. The interaction between year and treatment was statistically significant (*P* < 0.05) for soil enzyme activity (urease, acid phosphatase and sucrase) (**Supplementary Table S1**). Soil urease activity of the fertilizer treatments in 2020 was significantly lower than that of CK. Compared with CF and SNF2, SNF1 enhanced urease and acid phosphatase activity (**Figures 2A, B**), the urease increased by 2.29 times and 1.73 times, respectively (**Figure 2A**). In 2021, the urease activity of CF was significantly higher than that of CK and SNF1, increasing by 1.19 times and 2.16 times, separately (**Figure 2A**). The reason for decrease of urease activity in SNF1 might be the difference in nutrient content and form among treatments. The correlation results also found that urease activity was significantly positively correlated with Nmin, pH and SOM, and significantly negatively correlated with Mg^2+^, K^+^, Ca^2+^, and AP. Contrarily, sucrase activity was significantly negatively correlated with Nmin, pH, and SOM, and significantly positively correlated with Mg^2+^, K^+^, and Ca^2+^ (**Supplementary Figure S1A**). The sucrase activity of CF in 2020 and 2021 was significantly higher than that of SNF1 and SNF2 (**Figure 2B**). It could be concluded that soil chemical agrochemical properties and enzyme activities would be significantly regulated by the SNFert application.

### 3.3 Effect of SNFert on soil microbial diversity

**Figure 3:**
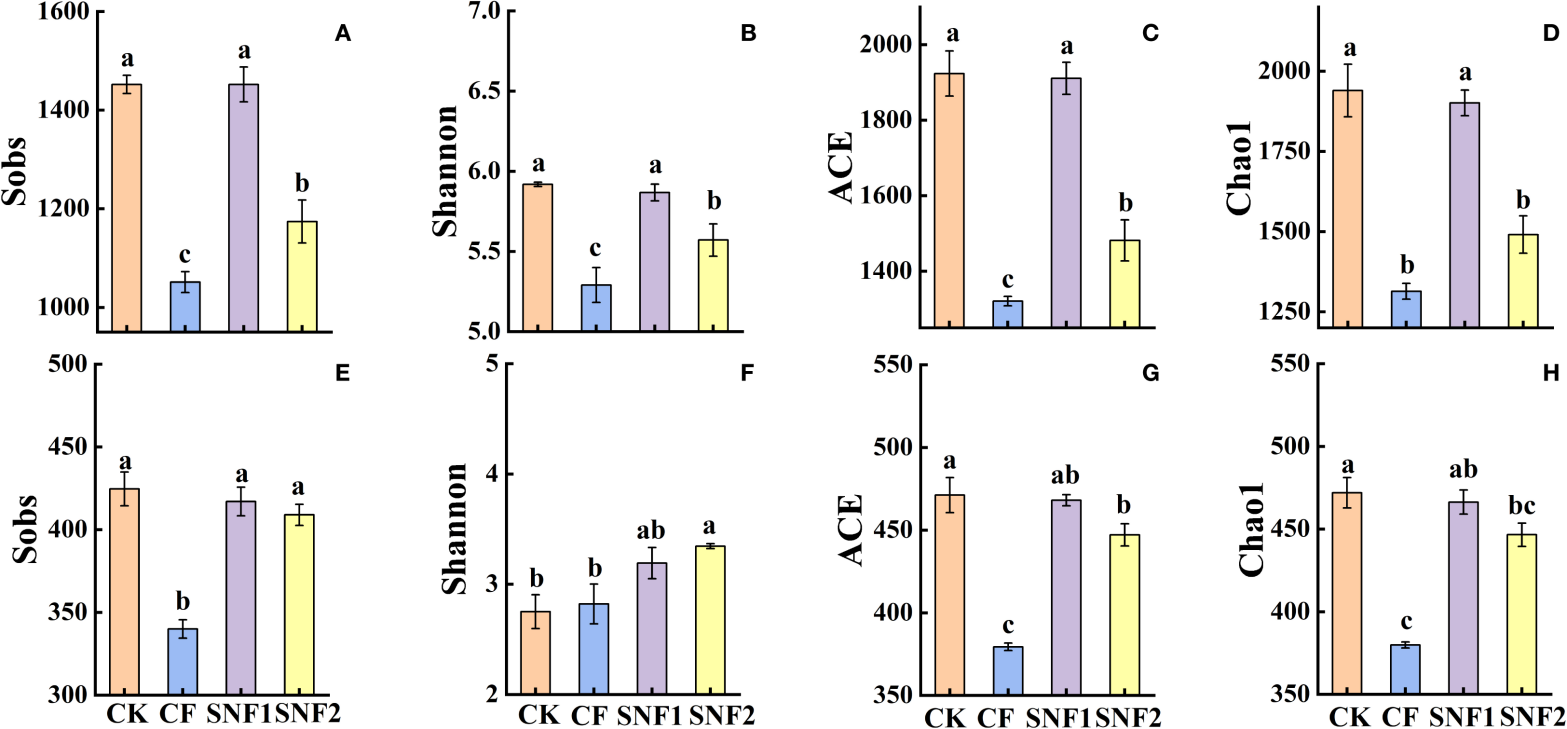
Sobs **(A, E)**, Shannon **(B, F)**, ACE **(C, G)**, Chao1 **(D, H)** index of alpha diversity of bacteria and fungi under SNFert in 2020. CK, CF, SNF1 and SNF2 stand for non fertilizer, conventional fertilizer, 15% less than CF and 20% less than CF, respectively. Different lowercase letters above bars in the same year indicate significant differences between the four treatments (*P* < 0.05, n = 3).

**Figure 4:**
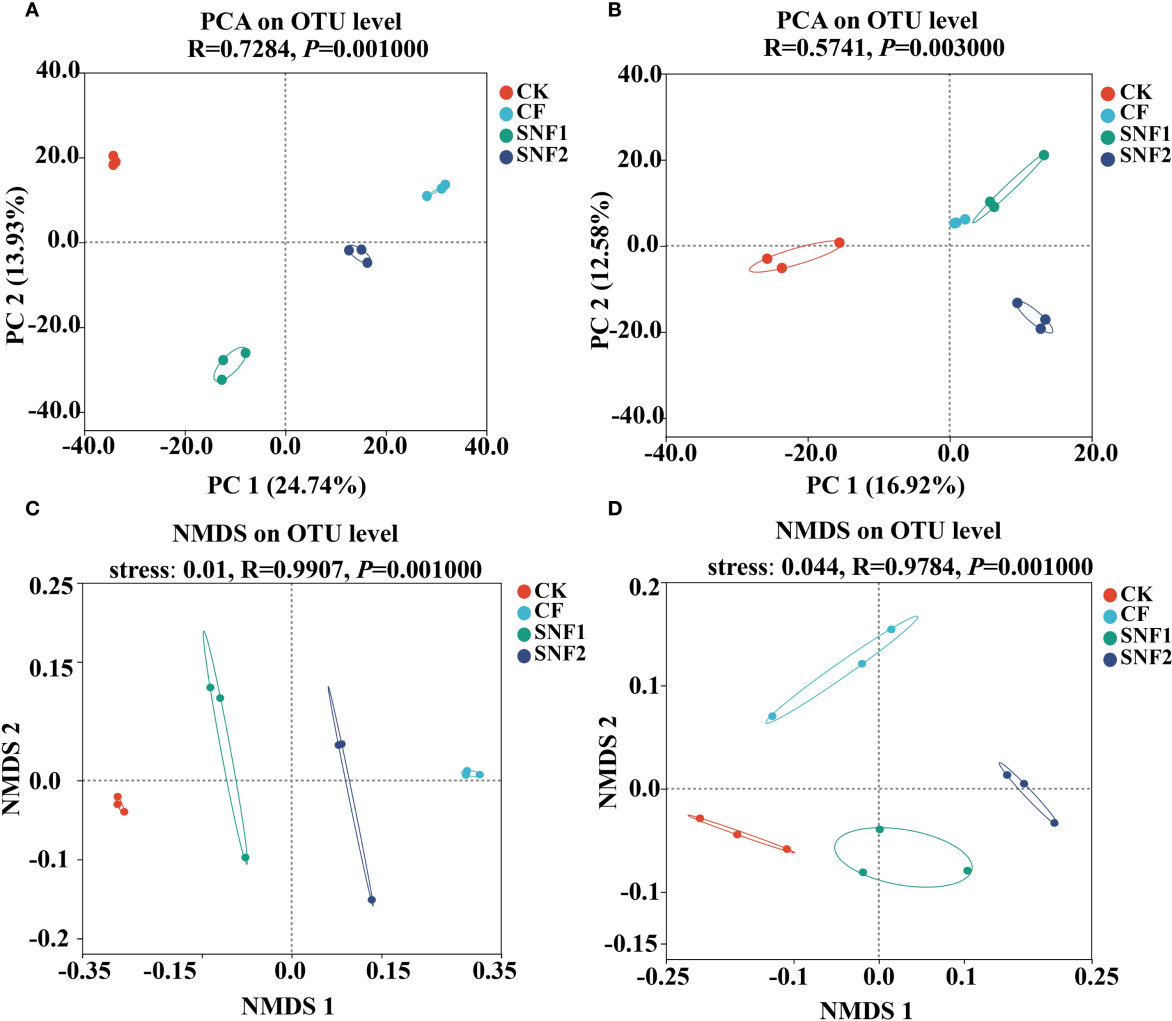
Principal components analysis (PCA) of bacterial **(A)** and fungi **(B)** in 2020. Non-metric multidimensional scaling (NMDS) analysis of bacteria **(C)** and fungi **(D)** in 2020. The larger the distance between treatments in the PCA plot, the greater the difference in the composition of species between treatments, and vice versa. CK, CF, SNF1 and SNF2 stand for non fertilizer, conventional fertilizer, 15% less than CF and 20% less than CF, respectively.

Based on established evidence, the first year (2020) was a critical period for altering soil microbial diversity after applying SNFert (Su et al., 2025), whereas subsequent years might remain stable (Fox et al., 2022). Consequently, the soil microbial diversity in the critical period (2020) was analyzed in the study. The results showed that the alpha and beta diversity of soil microorganisms were significantly affected by fertilizers (**Figures 3**, **4**). **Figure 3** showed that the Sobs, ACE and Chao1 indices of soil fungi and bacteria were the highest in the CK treatment. The consistent pattern was that bacterial diversity indices (Sobs, Shannon, ACE and Chao1) in SNF1 showed no significant difference from CK (*P*>0.05), yet significantly greater than those of CF (*P*<0.05) (**Figures 3A–D**). Although the Sobs, Shannon, ACE and Chao1 indices of SNF2 were lower than those of CK, they remained significantly higher than those of CF (*P*<0.05) (**Figures 3A–D**). Therefore, the SNFert application was more beneficial to soil microbial diversity than CF. Results indicated that the Sobs, ACE and Chao1 indices of fungi in SNF1 were not significantly different from those of CK and SNF2 (*P*>0.05), but were significantly higher than those of CF (*P*<0.05) (**Figures 3E, G, H**). Collectively, SNFert application, especially SNF1, could overcome the shortcomings of CF in reducing the alpha diversity of soil microorganisms.

Whether bacteria (**Figure 4A**) or fungi (**Figure 4B**), the distances between the three soil samples in the same treatment were close and no difference in the PCA plot, indicating that the species composition of the same treatment was similar. However, the distance between each treatment was significantly different, indicating distinct community structures of bacterial (**Figure 4A**) and fungal (**Figure 4B**) (*P*<0.01) among all treatments. Consistent with this, the NMDS indicated that there was a significant difference in the microbial community structure of bacterial (*P* < 0.01, R = 0.9907) and fungal (*P* < 0.01, R = 0.9784) among all treatments (**Figure 4C, D**). In other words, the bacteria and fungi of each treatment belong to a separate microbial community. Thus, SNFert, especially SNF1, could maintain the alpha diversity of soil bacteria and fungi at the CK level (*P*>0.05) (**Figure 3**), each treatment had its own bacterial and fungal communities (**Figure 4**).

### 3.4 Effect of SNFert on composition of microbial community

**Figure 5:**
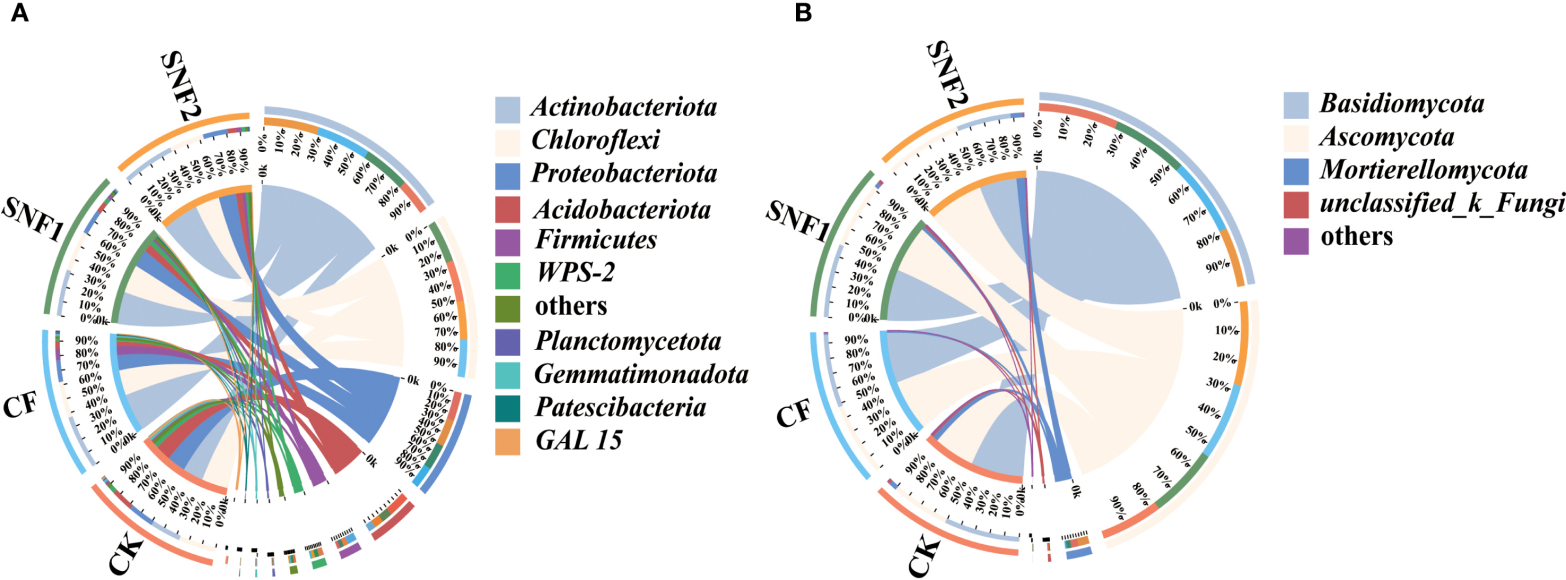
Relative abundance of soil bacterial **(A)** and fungi **(B)** phylum in 2020 (n = 3). The plot is divided into two parts. The rightmost one showed the abundance of dominant microbial species, while the leftmost part represented treatments in the study. The width of each ribbon in the plot represents the abundance of dominant species in each treatment. “Others” represents the set of species whose abundance ratio is less than 0.01 in all treatments CK, CF, SNF1 and SNF2 stand for non-fertilizer, conventional fertilizer, 15% less than CF and 20% less than CF, respectively.

The changes of initial microbial community (bacterial and fungal taxa) in 2020 at the phylum level were analyzed (**Figure 5**; **Supplementary Table S2**). The top 5 bacterial phyla and top 3 fungal phyla based on average relative abundance greater than 5.00% were selected. For bacteria, the dominant phyla in each treatment were *Actinobacteriota*, *Chloroflexi*, *Proteobacteria*, *Acidobacteriota* and *Firmicutes* (**Figure 5A**). For fungi, the dominant phyla were *Basidiomycota*, *Ascomycota* and *Mortierellomycota* (**Figure 5B**). However, the relative abundance of the same dominant species in each treatment was different, as shown on the left side of **Figure 5A, B**. The relative abundance of *Chloroflexi*, *Proteobacteria* and *Acidobacteriota* in the SNFert was significantly higher than those in CF (**Supplementary Table S2**). Among them, the abundance of the three bacteria in SNF1 was 28.79%, 17.75% and 8.09%, separately (**Figure 5A**). In the fungi composition, the relative abundance of *Mortierellomycota* in SNF1 increased compared with CF (**Figure 5B**; **Supplementary Table S2**). The relative abundance of *Ascomycota* (53.03%) and *Mortierellomycota* (8.23%) was increased in SNF2. In conclusion, SNFert could significantly increase the proportion of beneficial functional bacteria and fungi when CF was replaced with SNFert.

**Figure 6:**
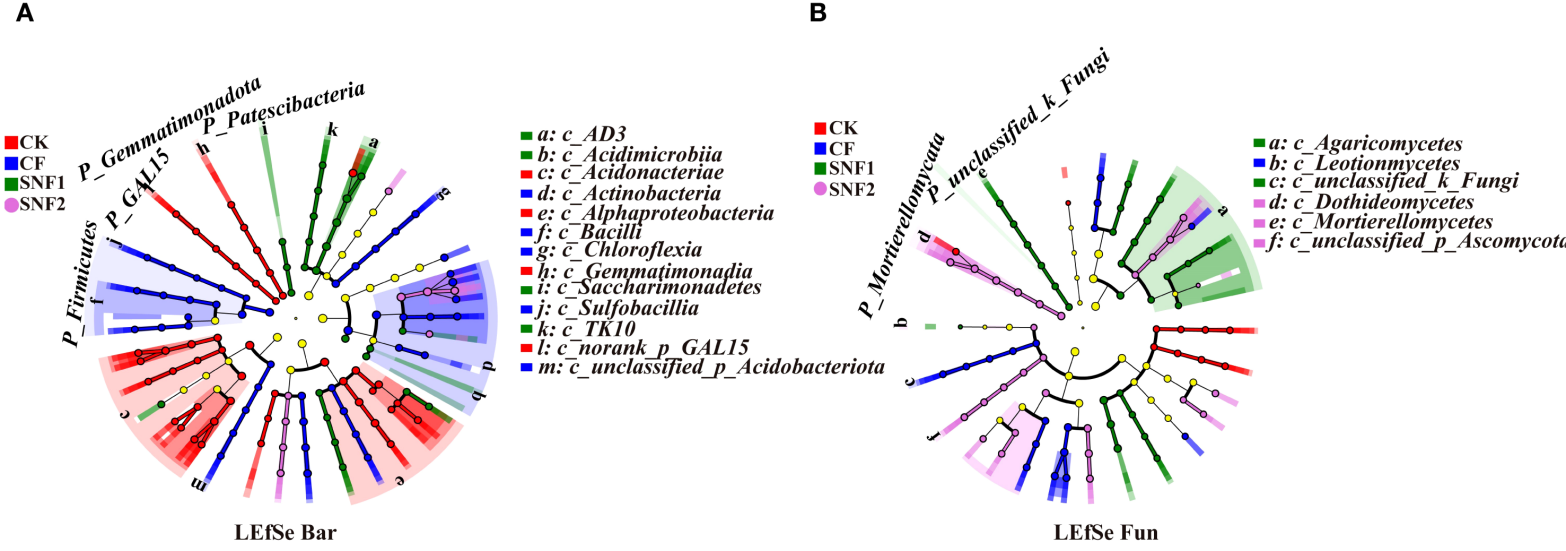
Linear discriminant analysis effect size (LEfSe) of microbial communities in 2020 (LDA score >3.5; *P* < 0.05, n = 3). LEfSe of bacteria **(A)** and fungal **(B)** from phylum-circles that are equidistant from the center outward represent the same classification level. The size of the nodes indicates the relative abundance of the species. The nodes of different colors represent the microbes that perform a crucial role in the grouping illustrated in the color, and yellow nodes denote non-significant (*P*>0.05) CK, CF, SNF1 and SNF2 stand for non-fertilizer, conventional fertilizer, 15% less than CF and 20% less than CF, respectively.

LEfSe identified microbial taxa (biomarkers) that play a key role in the environment in each treatment (LDA>3.5; *P* < 0.05, n = 3). LEfSe analysis in 2020 showed 90 biomarkers differed significantly among different treatments (**Figure 6**; **Supplementary Table S3**). The 32 taxa in the CK treatment, 33 taxa in the CF, 19 taxa in the SNF1, and 6 taxa in the SNF2 (**Supplementary Table S3**). Among them, *Gemmatimonadota*, *Firmicutes*, and *Patescibacteria* were significantly enriched in CK, CF and SNF1 treatment, and were key species. Similarly, a total of 58 fungal biomarkers were identified from the fungal taxa (**Supplementary Table S3**). The SNF1 had a higher number of enriched taxa (22), followed by the SNF2 (19). Notably, *Kickxellomycota*, *unclassified_k_Fungi* and *Mortierellomycota* were the key unique microbial taxa for CK, SNF1 and SNF2 treatment (**Figure 6B**). In summary, application of the SNFert could alter the soil microbial taxa that play a key role in soil environmental change. Consequently, the SNFert may affect the microbial function of sugarcane rhizosphere soil.

### 3.5 Effect of SNFert on microbial function

**Figure 7:**
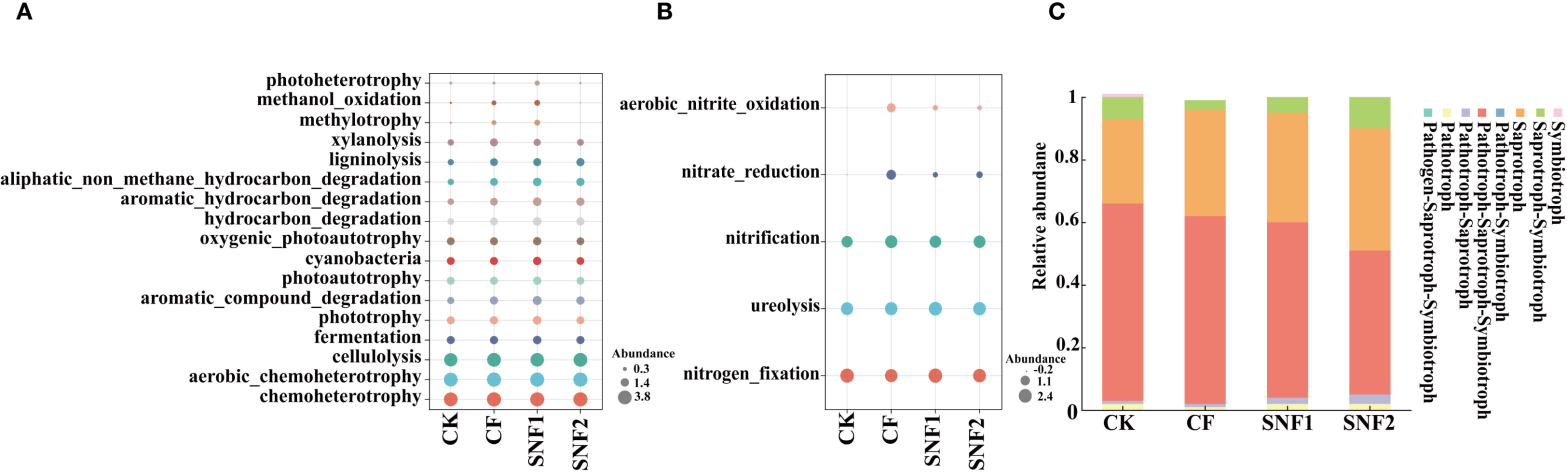
Predictive plots of the FAPROTAX of bacterial function (A and B) and FUNGuild of fungal function **(C)** in 2020 (n = 3). **(A, B)** are the bacteria species abundance involved in soil carbon and nitrogen cycle, respectively. The different colors in the bubble plots indicate different bacterial species. The diameter of each bubble indicates the relative abundance of the bacterial species. **(C)** is the relative abundance of fungal species in each treatment in different nutrient types. CK, CF, SNF1 and SNF2 stand for non-fertilizer, conventional fertilizer, 15% less than CF and 20% less than CF, respectively.

As shown in **Figure 7**, the microbial function was altered after soil microbial communities were optimized during the first growing season (2020). Compared with CK, each fertilization treatment could significantly increase the bacterial abundance of chemoheterotrophy, aerobic_chemoheterotrophy and cellulolysis, with the highest abundance observed in CF treatment. The species abundance of functions, including hydrocarbon_degradation, aromatic_hydrocarbon_degradation, photoheterotrophy and aromatic_compound_degradation in the SNF1 was higher than those of CF. The species abundance of these functions in SNF1 were increased by 71.43%, 71.43%, 112.50% and 62.07%, respectively (**Figure 7A**). It could be seen that the SNFert alleviated the effect of fertilizer on the functional bacteria in soil carbon cycling, and made the microbial function abundance more similar to CK treatment. In the soil nitrogen cycle, compared to CF, the species abundances of functions (nitrification, nitrate reduction and aerobic nitrite oxidation) in the CK and SNFert treatment were markedly decreased (**Figure 7B**). This indicated that the SNFert application could reduce nitrification and denitrification in soil. The species abundances of functions including nitrogen_fixation and ureolysis in SNF1 were increased by 16.80%–174.82% and 40.84%–114.24%, respectively, compared with the CK, CF and SNF2 treatment (**Figure 7B**). Therefore, the SNFert would promote soil carbon and nitrogen metabolism by mediating the abundance of carbon and nitrogen cycling functional bacteria.

A total of 8 nutrient types were identified by FUNGuild (**Figure 7C**). The dominant types were Pathogen-Saprotroph-Symbiotroph, Saprotroph and Saprotroph-Symbiotroph in CK, CF, SNF1, and SNF2 treatment, with a relative abundance of 46.28%–63.15%, 26.59%–38.91% and 3.00%–10.18%, respectively. The relative abundance of Saprotroph and Saprotroph-Symbiotroph species in SNFert treatments were notably higher than those of CF. In Pathogen-Saprotroph-Symbiotroph, the relative abundance of SNFert treatments was significantly lower than CF. Therefore, the SNFert could impact microbial function by regulating the proportion of soil microbial functional flora, which might be related to the change of sugarcane rhizosphere environment by the SNFert.

### 3.6 Correlation between soil microbial community structure & function and soil agrochemical properties

**Figure 8:**
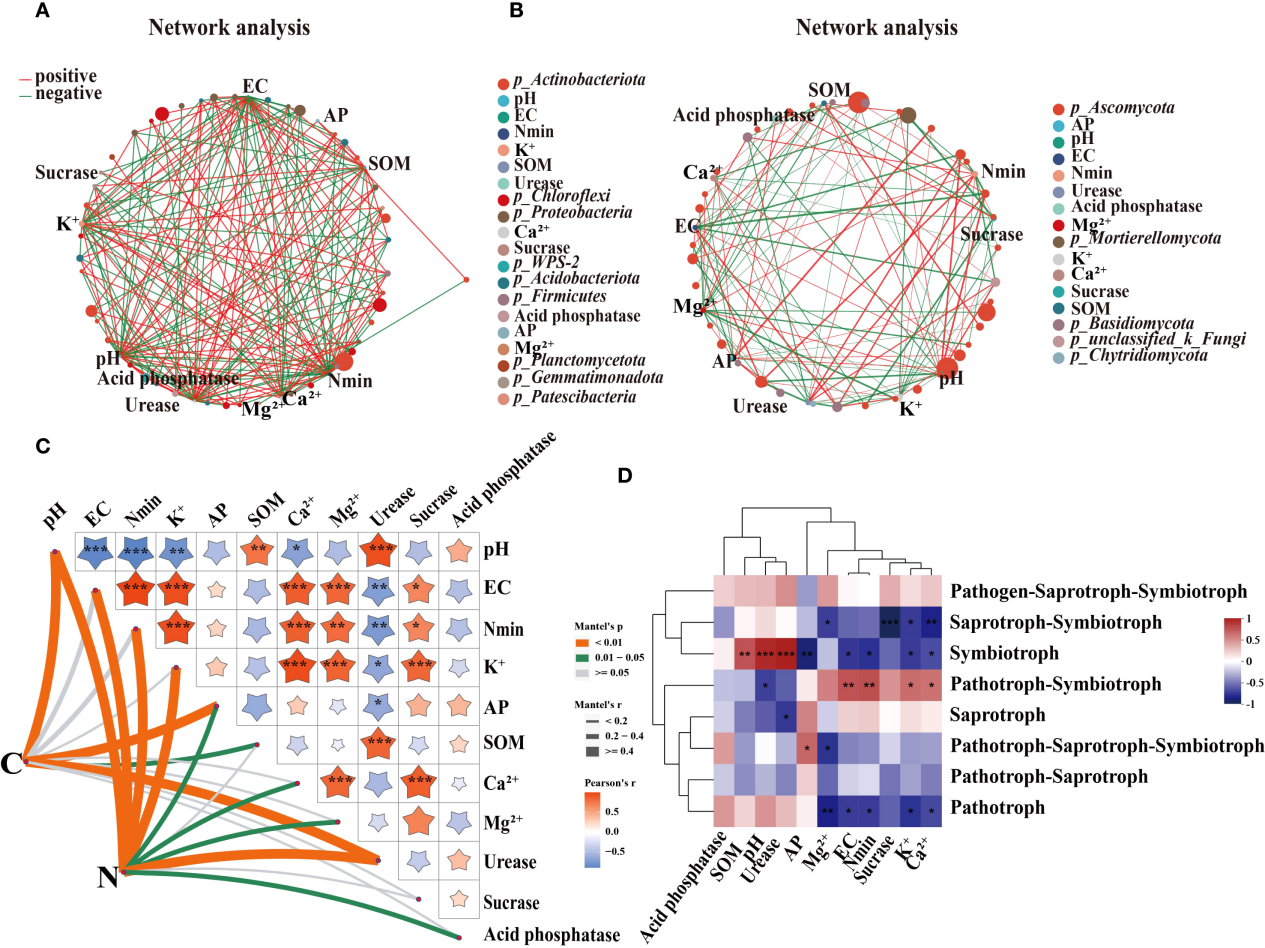
The correlation analysis of microbial communities and function with soil agrochemical properties. **(A)** and **(B)** are the two-factor correlation networks of bacterial and fungal communities with the soil agrochemical properties in 2020. The size of the node represents the abundance of each species. Different colors stand for different species. The correlation is represented by the color connection lines. The red color indicates a positive correlation. Green indicates a negative correlation. The thicker the line, the higher the correlation between species and factors. **(C)** Mantel analysis of bacteria function and the soil agrochemical properties in 2020. The right upper of **(C)** shows the Pearson correlation and the left bottom of **(C)** shows the Mantel analysis between the bacteria function and the soil agrochemical properties. The width and color of the lines show different Mantel’s r and *P*-values. **(D)** is the correlation heatmap between the function of fungi and the soil agrochemical properties. “*”, “**” and “***” mean significant correlation at 0.05, 0.01 and 0.001 levels, respectively.

Both soil microbial community structure and function were mainly influenced by pH (**Figure 8**; **Supplementary Table S4**). There was a significant positive correlation between pH and most bacteria in soil (**Figures 8A, B**). The minor factors that affected the soil microbial community were EC, Nmin, K^+^ and urease activity. The redundancy analysis (RDA) confirmed further that soil pH, EC, Nmin, K^+^ and urease activity had significant effects on the community composition of bacteria and fungi (**Supplementary Figure S2**).

Mantel analysis showed that the abundance of species involved in soil carbon and nitrogen cycling was significantly affected by soil pH, AP, and urease activity (**Figure 8C**). The soil carbon cycle was also positively correlated with SOM. **Figure 8C** showed that EC, Nmin, K^+^, Ca^2+^ and Mg^2+^ significantly affected soil nitrogen cycling. Furthermore, EC, Nmin, K^+^, Ca^2+^ and Mg^2+^ were significantly negatively correlated with the Symbiotroph and Pathotroph, and positively correlated with Pathotroph-Symbiotrop. The Symbiotroph was noticeably positively correlated with pH, SOM, urease activity (**Figure 8D**). Therefore, the soil microbial community structure and function could be regulated by SNFert through changing key soil factors.

### 3.7 Biotic and abiotic drivers of sugarcane yield

**Figure 9:**
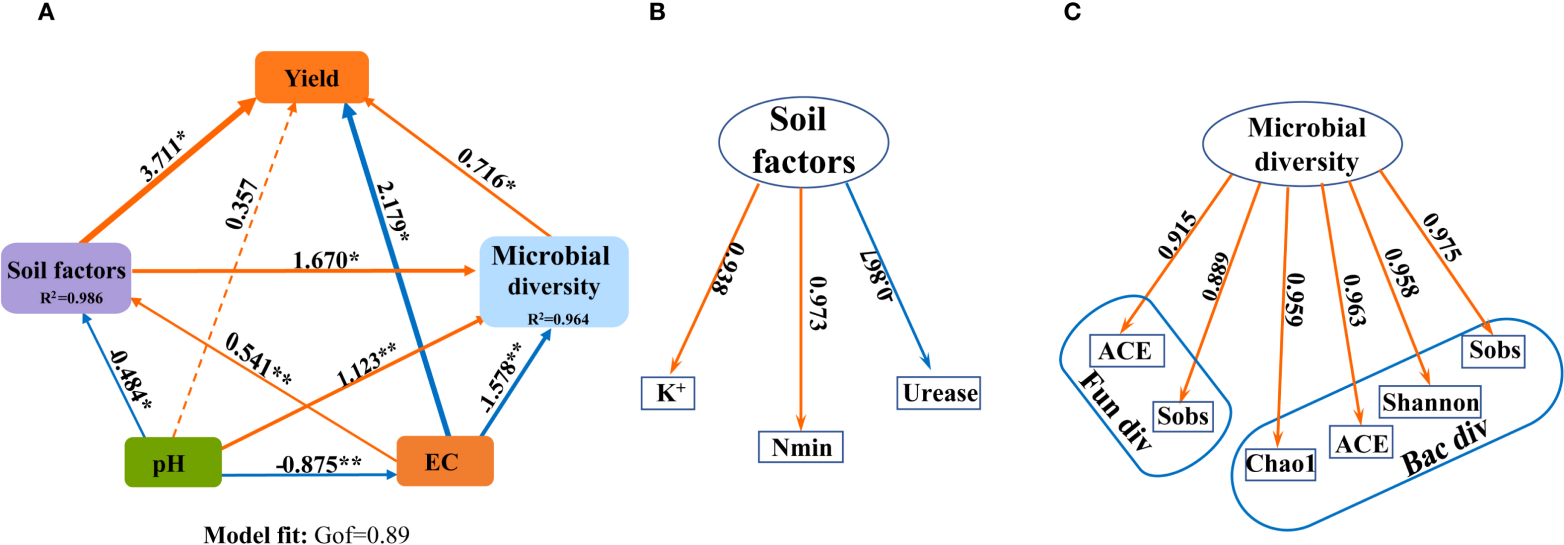
The effects of biotic and abiotic factors on the sugarcane yield. **(A)** is the partial least squares path models (PLS-PM) plot. **(B)** is the components of the Soil factors in PLS-PM (Nmin, K^+^ and urease activity in 2020). **(C)** is the component of the Microbial diversity in PLS-PM (bacterial and fungi alpha diversity indices in 2020). The orange and blue arrows indicate the positive and negative effects of causality, respectively. The numbers above the arrows indicate the direct effects coefficients. “*” and “**” mean significant correlation at 0.05 and 0.01 levels, respectively. R^2^: Measure the predictive ability of each latent variable in the model. The GOF is 0.89, which conforms to the standard of GOF > 0.7 in the model fitting parameters.

A partial least squares path model (PLS-PM) was constructed to explore the complex relationship among the soil agrochemical properties, microbial diversity, yield under the SNFert application during the first growing season (2020) (**Figure 9**). **Figure 9** showed that pH, EC, Soil factors and microbial diversity had a direct effect on sugarcane yield, with path coefficients of 0.357, -2.179, 3.711 and 0.716, respectively (**Figure 9**). The correlation research results also indicated that sugarcane yield was significantly negatively correlated with soil pH and urease activity. The microbial diversity indices (Sobs, ACE, Chao1) were significantly positively correlated with soil pH and urease activity, and significantly negatively correlated with Nmin and EC (**Supplementary Figure S1B**). This indicated that the SNFert might directly affect sugarcane yield by regulating soil agrochemical properties and microbial diversity. Soil pH had both positive and negative indirect effects on sugarcane yield through EC, Soil factors, or Microbial diversity. Specifically, sugarcane yield was influenced by following seven pathways: pH–EC–yield, pH–EC–Soil factors –yield, pH–EC–Soil factors–Microbial diversity–yield, pH–EC–Microbial diversity–yield, pH–Soil factors–yield, pH–Soil factors–Microbial diversity–yield and pH–Microbial diversity–yield. The corresponding indirect effect coefficients were 1.907, -1.757, -0.566, 0.989, -1.796, -0.579 and 0.804, separately. Notably, pH had a significant negative effect on EC, and a direct positive effect on Microbial diversity (**Figure 9**). This suggested that the higher the soil pH, the lower the EC value, the more conducive to microbial development. Therefore, the SNFert application was able to increase sugarcane yield by alleviating soil pH, reducing soil nutrient residues and EC values, and increasing soil microbial diversity.

## 4 Discussion

### 4.1 Mechanism of increasing yield and improving soil with SNFert

Up to now, most studies on the effect of synchronized nutrition fertilizer on field crops in north China have focused on yield response (Wang et al., 2019; Andrade et al., 2021; Li et al., 2022). Similarly, this study demonstrated that SNFert application, even with 15% to 25% less fertilizer input, could achieve the same sugarcane yield and sucrose content as CF in two years (**Table 3**). The possible reason is that the SNFert could meet the nutrient demand even with a single application compared to CF, owing to its composition of fast-acting, long-acting and control-acting nutrients (Wei et al., 2021). The maintenance of sugarcane yield under reduced fertilization input was attributed to SNFert increasing the number of tillers and effective stalks of sugarcane (**Table 3**). Because the SNFert was also characterized by slow release and supply of nutrients, it could prevent losses of the nutrients and increase fertilizer utilization efficiency (Wei et al., 2021; Li et al., 2022). Research has found that slow-release nutrients in CRFs could effectively reduce the excessive accumulation of soil nutrients, which was an effective measure to prevent soil pH decrease, improve N utilization efficiency, and increase crop yield (Wu et al., 2022; Lü et al., 2023; Deng et al., 2023). Specifically, the soil pH in the low nitrogen amount of SNFert treatments was significantly higher than that of the CF treatment (**Figure 1H**). The results of the study also found that effect of the SNFert on soil pH was influenced by precipitation. Extreme and continuous rainfall during fertilization (http://www.lax.gov.cn/zt/qxj_qxfwxx/t4678025.html, 2021) would trigger nutrient runoff and leaching (Wang et al., 2023), resulting in Nmin depletion and EC reduction (**Figure 1**), subsequently altering pH and enzyme activity (**Figure 2**). In this case, lower EC means depletion of nutrients in the soil. This study found that the EC of SNF1 was higher than that of CF in 2021 (**Figure 1G**), which indicated that SNF1 could still provide sufficient nutrients for sugarcane growth under unnormal weather conditions. This was because the SNFert, as a new type of CRF, is made of controlled-release NPK compound, which attenuate rainfall-induced nutrient losses through regulated release kinetics (Zhao et al., 2022). Nevertheless, EC of SNF1 in 2021 was still lower than the EC of CF in 2020 (0.72 dS·m^-1^) (**Figure 1G**). The research found that the EC threshold that affected microbial metabolic processes was 0.70 dS·m^-1^ (Yan et al., 2015). That was to say, when the EC value was higher than 0.70 dS·m^-1^, the microbial metabolic processes in the soil would be restricted. The significant negative correlation between EC and urease activity also proved this point (**Supplementary Figure S1A**). From this, the application of SNFert not only was able to provide enough nutrients for sugarcane even under heavy rainfall condition, but also ensured the normal microbial metabolic processes under the condition.

Similarly, compared to CF, SNF1 significantly enhanced urease activity in 2020 (**Figure 2A**). Urease is a key rate-limiting enzyme in nitrogen mineralization processes, and an increase in its activity can accelerate N mineralization in the soil (Du et al., 2018). However, it was worth noting that SNF1 reduced urease activity in 2021 (**Figure 2A**). Generally, soil enzyme activity is closely associated with microbial activity (Bellotti et al., 2022). Under waterlogging conditions, microbial activity could be inhibited, leading to decreased urease activity (Bueis et al., 2018). Correlation analysis revealed a significant positive relationship between soil pH and urease activity (**Supplementary Figure S1A**). The decrease in soil pH limited *ureC* microbial diversity, which was the main limiting factor for soil urease activity (Feng et al., 2025). Therefore, the inconsistencies between years in urease activity might be attributed to the slow nutrient release from SNF1 under heavy rainfall conditions, which reduced soil pH. In contrast, sucrase activity in 2021 was higher than that in 2020, which was similar to the findings of Chen et al. (2024). The possible reason was that soil mineral nitrogen content in CF was reduced by heavy rainfall, which intensified competition for nutrients between sugarcane roots and soil microorganisms, thereby promoting root exudate production (Sugihara et al., 2010; Chen et al., 2024). This interpretation was supported by a significant negative correlation between Nmin content and sucrase activity (**Supplementary Figure S1A**). Moreover, the increase in soil moisture due to rainfall could enhance the mobility of enzyme substrates and provide a suitable environment for enzymatic reactions (Bandyopadhyay et al., 2010). Furthermore, higher nitrogen application rates in the CF treatment accelerated soil nutrient turnover rate (Chen et al., 2024), resulting in an increase in soil sucrase activities in CF in two years.

**Figure 10:**
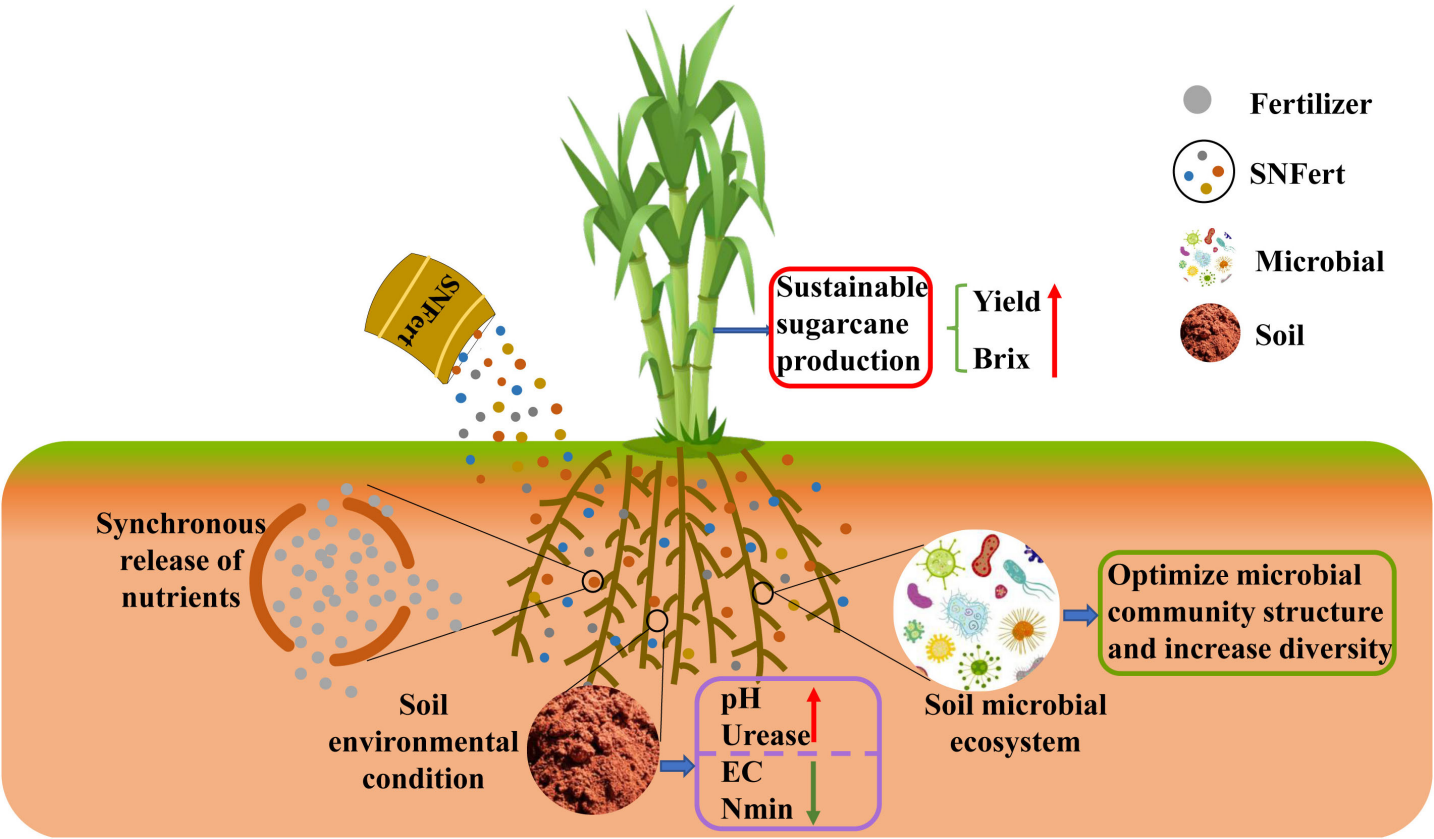
Mechanism of SNFert improved sustainable sugarcane production.

Remarkably, the present research in 2020 also confirmed that the SNFert enhanced sugarcane yield through its dual capacity to alleviate soil acidification and increase microbial diversity (**Figures 9**, **10**). Preventing soil acidification would increase soil enzyme activity and soil microbial diversity, thereby maintaining sugarcane productivity (Li et al., 2022; Liu et al., 2022; Deng et al., 2023). Numerous studies have found that increasing soil microbial diversity improved soil nutrient availability and plant productivity (Delgado-Baquerizo et al., 2020; Sekulić et al., 2023). Similarly, PLS-PM revealed that the yield of 2020 gains correlated with microbial diversity improvement (**Figure 9**). Ji et al. (2022) suggested that increasing microbial diversity and community complexity could significantly improve the nitrogen agronomic efficiency of maize. This might be because more complex microbial communities could accelerate soil nutrient cycling processes and enhance plant-microbial responses (Fan et al., 2020). Liang et al. (2017) and Qiu et al. (2021) also reported that increasing soil microbial multifunctionality (nutrient cycling) could increase soil nutrient content, thereby increasing crop yield. It was worth noting that although microbial data were not collected in 2021, the consistent trends in soil properties (e.g., pH, SOM) and yield between the two years suggested that the response of microbial diversity to SNFert might have been consistent across years. This perspective aligned with Li et al. (2021), who described the succession of microbial communities and functions as characterized by a long-term nature, stability and continuity. Therefore, we inferred that the soil environment in the SNFert treatments was conducive to the optimization of soil microbial communities and function, which was beneficial to soil nutrient cycling and sugarcane yield. These findings represented novel insights into the beneficial function of the SNFert application in sugarcane fields. Notwithstanding, the lack of microbial data in 2021 was still a limitation. Long-term microbial shifts should be evaluated beyond one season in the future research.

### 4.2 Mechanism of microbial diversity increased by the SNFert

Based on the immediate response of microbial diversity to the application of SNFert (Han et al., 2023; Su et al., 2025), the first year (2020) after applying SNFert was a critical period for analysing microbial diversity. The results in 2020 indicated that SNFert mitigated the negative impact of CF on soil microbial alpha diversity (Sobs, Shannon, Ace, and Chao), as evidenced by microbial diversity under SNFert remaining at the control level (**Figure 3**). This result was similar to that of Ji et al. (2022), where combined application of polymer-coated urea (PCU) and conventional urea could significantly increase the soil microbial diversity indices at the later stage of maize growth. This increase in diversity might be attributed to two factors. First, the SNFert was rich in a variety of nutrients, which directly provided abundant nutrients for soil microbes (Soumare et al., 2023). Second, the SNFert application would promote root number, root length, root surface area and root activity of the sugarcane (Jiang et al., 2022). A developed root system meant more root secretions and exfoliations, which could provide sufficient carbon sources for the soil microbial growth (Li et al., 2021), thus increasing the microbial abundance and diversity. Contrastingly, the Shannon index of bacteria and fungi in PCU treatment was lower than that in CF (Sun et al., 2023). The possible reason was that the higher total nitrogen application and slower nitrogen release rate from PCU in the whole period of wheat growth, resulted in a high accumulation of NO_3_^-^ and NH_4_^+^ in the wheat maturation stage (Sun et al., 2023). Zhang et al. (2018) noted that most beneficial bacteria could not withstand the high salinity caused by excessive CF. Higher soil nitrogen and salinity would destroy soil microorganisms (Li et al., 2021; Ge et al., 2025). The correlation results also confirmed this point that soil EC and Nmin were significantly negatively correlated with soil microbial diversity indices (*P* < 0.05). The high content of available nutrients in the soil led to a decrease in soil pH (Tang et al., 2022). Lower pH reduced the suitability of most microbial habitats and promoted the colonization of a few acid-resistant and acid-producing microorganisms, thereby reducing microbial diversity (Chen et al., 2016; Hottenstein et al., 2019). The significant positive correlation between pH and microbial diversity observed in 2020 further proved this point in reverse. From this, SNFert could enhance soil microbial diversity by improving the soil conditions for soil microbial growth (**Figure 10**).

The change in soil microbial diversity was due to the change in soil microbial species or quantity (Navarro-Noya et al., 2021). PCA and LEfSe analyses in 2020 revealed that there was a separate microbial community in the SNFert treatments (**Figures 4**, **6**), suggesting that the SNFert altered microbial community structure. This might be due to their different survival strategies (Sessitsch et al., 2001). The changes of soil nutrients might selectively promote or inhibit the growth of soil microbial populations with different nutrient requirements. For example, *Proteobacteria*, as a key species in the microbial health network system (Yang et al., 2017), were known as fast-growing co-trophic organisms with high nutritional requirements (Saleem et al., 2016). *Chloroflexi* and *Acidobacteriota*, typical dominant oligotrophic types of phyla in the sugarcane rhizosphere bacterial community, were more likely to survive in environments with lower nutrients (Lian et al., 2019; Wu et al., 2023; Wang et al., 2022). In this study, the SNFert increased the abundance of key functional species during the first year of fertilization, such as *Chloroflexi*, *Proteobacteria*, *Acidobacteriota*, *Basidiomycota* and *Mortierellomycota* (**Figures 5**, **6**). Studies found that the abundance of *Acidobacteria* and *Proteobacteria* showed an opposite increasing trend under the condition of high nutrition (Song et al., 2023). Conversely, the SNFert increased the species abundance of both co-trophic and oligotrophic types. This indicated that the SNFert reduced competitive exclusion between species and increased the species abundance of more ecological niches through controlling nutrient release (Delgado-Baquerizo et al., 2020). It is worth noting that although this model indicates the existence of mutually beneficial relationships among microorganisms, direct verification through the microbial species correlation network analysis is still indispensable.

FAPROTAX prediction in 2020 indicated that SNFert attenuated the adverse effects of CF on functional bacteria in soil carbon and nitrogen cycling. Specifically, SNFert increased the abundance of functional bacteria in hydrocarbon degradation, nitrogen fixation and ureolysis, and reduced the abundance of bacteria involved in nitrification and denitrification (**Figures 7A, B**). This was similar to the findings of Gao et al. (2023). This functional shift was driven by the SNFert regulation of the dominant taxa abundance involved in the soil carbon and nitrogen cycle. Specifically, *Chloroflexi* exhibited diverse trophic modes, performed both aerobic and anaerobic respiration, could generate energy through photosynthesis, and participated in C and N cycles (Ehrich et al., 2007). *Proteobacteria* participated in soil phosphate solubilisation and nitrogen fixation, which could enhance plant disease resistance and promote plant growth (Wei et al., 2023). *Acidobacteriota*, *Basidiomycota* and *Mortierellomycota* in the SNFert treatments facilitated soil carbohydrate degradation, inorganic carbon fixation and organic carbon synthesis, respectively (Larsbrink and McKee, 2020; Luo et al., 2023). Coordinated improvements in C and N cycling functions suggested that SNFert might have the potential to enhance microbial multifunctionality (Liang et al., 2017). Meanwhile, the soil microbial function optimization by the SNFert also indicated that appropriate nitrogen reduction could improve the microbial adaptability to the soil environment, and minimize N_2_O emissions and nitrate leaching risks. This will need to be further validated by measuring nitrogen-related indicators such as total N recovery or nitrogen use efficiency (NUE) indicators, N_2_O emissions, etc. FUNGuild analysis in 2020 further revealed that compared with CF, the Saprotroph-Symbiotroph population increased by 60.91%–240.68% under the SNFert (**Figure 7C**), indicating that SNFert might facilitate improved plant-microbe nutritional exchanges. This was critical for nutrient utilization and plant disease suppression (Khan et al., 2023). Nevertheless, the limitation of this study was that microbial data only focused on the initial fertilization year (2020), which limited to assess interannual variability. In addition, continuous monitoring of microbial dynamics in multi-seasons would be necessary in the future to validate the long-term effect of SNFert on the soil microbial community.

## 5 Conclusions

The study revealed that synchronized nutrition fertilizers (SNFert) significantly altered the soil agrochemical properties when CF was replaced with SNFert. SNFert significantly reduced the accumulation of salts such as Nmin, H_2_PO_4_^-^, K^+^ in soil, and increased soil pH to prevent or alleviate soil acidification. An increase in soil pH, a decrease in Nmin and EC had offered suitable conditions for soil microbes to enrich soil microbial diversity. As a new type of fertilizer, after applying SNFert in the first year (2020), the negative impact of CF on the alpha diversity of soil microorganisms was alleviated. This was manifested in that the diversity of soil microorganisms in the SNFert-treated plots remained at the level of the control. The beneficial functional bacteria in soil microbial community were increased by application of the SNFert. The soil carbon and nitrogen cycle were promoted by those beneficial taxa. Therefore, the structure and function of soil microbial community could be regulated by the SNFert. There was the same trend of yield response to the SNFert in the two years and the yield of SNF1 treatment even increased by 6.83% in the second year. This result might support the consistency between microbial response and fertilizer efficiency across years. Therefore, under the SNFert application for two consecutive years, the sugarcane yield was influenced by the interaction between soil microbial diversity and soil agrochemical properties. In conclusion, use of SNFert in sugarcane cultivation will be an effective measure to reduce CF input and increase sugarcane yield because the SNFert is able to create a suitable soil agrochemical environment and increase soil microbial diversity.

## Data Availability

The data presented in the study are deposited in the Mendeley Data. This data can be found here: https://data.mendeley.com/datasets/z73xdcb6pd/1.
