# Supplementary material for: Synchronized nutrition fertilizer improves soil microbial diversity and sugarcane yield through agrochemical and enzymatic regulation
Source: Front Plant Sci. 2025 Oct 2;16:1694590. doi: 10.3389/fpls.2025.1694590 (PMC12527858; doi:10.3389/fpls.2025.1694590)
Supplement: Supplementary file 1 [file DataSheet1.docx]

Supplementary Material

# Supplementary Figures and Tables

## Supplementary Tables

**Supplementary Table 1.** Variance analysis of the effects of treatment (T), year (Y), and their interactions on the soil agrochemical properties

| Soil properties | Sources of variation | | | |
| --- | --- | --- | --- | --- |
|  | Year (Y) | Treatment (T) | Y×T | Total variance |
| pH | 0.5 ^ns^ | 3.0 ^ns^ | 475.3^**^ | 1.0 |
| EC (dS·m^-1^) | 0.1 ^ns^ | 1.4 ^ns^ | 1054.0^**^ | 0.9 |
| Nmin (mg·kg^-1^) | 2.8 ^ns^ | 1.0 ^ns^ | 477.4^**^ | 52888.3 |
| AP (mg·kg^-1^) | 4.6 ^ns^ | 12.6^*^ | 3.0 ^ns^ | 8749.3 |
| K^+^ (mg·kg^-1^) | 6.6 ^ns^ | 5.8 ^ns^ | 76.1^**^ | 551207.5 |
| Ca^2+^ (mg·kg^-1^) | 1.5 ^ns^ | 1.0 ^ns^ | 48.9^**^ | 37028.6 |
| Mg^2+^ (mg·kg^-1^) | 10.1^*^ | 1.2 ^ns^ | 76.8^**^ | 885.4 |
| SOM (g·kg^-1^) | 5.4 ^ns^ | 0.8 ^ns^ | 7.0^**^ | 91.6 |
| Urease (U·g^-1^·24h^-1^) | 4.9 ^ns^ | 1.0 ^ns^ | 572.9^**^ | 17231382.0 |
| Acid phosphatase (U·g^-1^·24h^-1^) | 205.2^**^ | 1.1 ^ns^ | 13.2^**^ | 342573.6 |
| Sucrase (U·g^-1^·24h^-1^) | 22.0^*^ | 0.7 ^ns^ | 74.6^**^ | 141618.7 |

The values shown are the *F*-statistic of the analysis of variance ((ANOVA). “*” and “**” mean significant correlation at 0.05 and 0.01 levels, respectively. “ns” mean not significant.

**Supplementary Table 2.** The relative abundance of soil microbial community in 2020 under different treatments

| Species name | | Treatments | | | |
| --- | --- | --- | --- | --- | --- |
|  |  | CK | CF | SNF1 | SNF2 |
| Bacterial | *Actinobacteriota* | 22.97±0.01b | 38.38±0.00a | 32.69±0.04a | 39.02±0.04a |
|  | *Chloroflexi* | 27.75±0.01a | 25.60±0.03a | 28.79±0.04a | 25.73±0.05a |
|  | *Proteobacteria* | 19.47±0.02a | 15.35±0.01a | 17.75±0.02a | 18.74±0.02a |
|  | *Acidobacteriota* | 16.34±0.01a | 5.48±0.01b | 8.09±0.01b | 6.67±0.02b |
|  | *Firmicutes* | 2.29±0.00b | 7.90±0.01a | 3.41±0.00b | 3.75±0.00b |
|  | *WPS2* | 4.47±0.00a | 1.61±0.00b | 3.70±0.01a | 2.61±0.01ab |
|  | *Planctomycetota* | 1.11±0.00a | 1.54±0.00a | 1.06±0.00a | 0.89±0.00a |
|  | *Gemmatimonadota* | 1.54±0.00a | 1.54±0.00a | 0.89±0.00b | 0.29±0.00c |
|  | *Patescibacteria* | 0.45±0.00c | 0.99±0.00ab | 1.16±0.00a | 0.80±0.00b |
|  | *GAL15* | 1.01±0.00a | 0.01±0.00b | 0.20±0.00b | 0.03±0.00b |
|  | *others* | 2.60±0.00a | 1.61±0.00c | 2.26±0.00b | 1.46±0.00c |
| Fungal | *Basidiomycota* | 52.07±0.04a | 48.99±0.05a | 49.14±0.03a | 37.55±0.01b |
|  | *Ascomycota* | 40.07±0.03b | 49.23±0.04ab | 45.16±0.03ab | 53.03±0.00a |
|  | *Mortierellomycota* | 5.59±0.01b | 1.25±0.00d | 3.30±0.01c | 8.23±0.01a |
|  | *Fungi* | 1.75±0.00a | 0.37±0.00b | 1.75±0.00a | 0.71±0.00b |
|  | *others* | 0.51±0.00ab | 0.16±0.00c | 0.65±0.00a | 0.48±0.00b |

CK, CF, SNF1 and SNF2 stand for non fertilizer, conventional fertilizer, 15% less than CF and 20% than CF. The numbers are mean ± standard error (SE, n = 3). Different lowercase letters in the same row indicate significant differences among the four treatments (*P* < 0.05).

**Supplementary Table 3.** The relative abundance of soil microbial community in 2020 under different treatments

| Species name | | Treatments | Mean | LDA_value | *P-*value |
| --- | --- | --- | --- | --- | --- |
| Bacterial | *p__Actinobacteriota;c__Actinobacteria;o__Frankiales;f__Acidothermaceae;g__Acidothermus* | SNF2 | 5.202 | 4.492 | 0.038 |
|  | *p__Acidobacteriota;c__Acidobacteriae;o__Subgroup_2;f__norank_o__Subgroup_2;g__norank_f__norank_o__Subgroup_2* | CK | 4.617 | 4.270 | 0.038 |
|  | *p__GAL15;c__norank_p__GAL15;o__norank_c__norank_p__GAL15;f__norank_o__norank_c__norank_p__GAL15* | CK | 4.006 | 3.718 | 0.018 |
|  | *p__Actinobacteriota;c__Acidimicrobiia* | SNF1 | 4.451 | 3.757 | 0.041 |
|  | *p__Proteobacteria;c__Alphaproteobacteria;o__Rhizobiales;f__Xanthobacteraceae;g__unclassified_f__Xanthobacteraceae* | CK | 4.170 | 3.763 | 0.019 |
|  | *p__Gemmatimonadota;c__Gemmatimonadetes;o__Gemmatimonadales;f__Gemmatimonadaceae* | CK | 4.165 | 3.845 | 0.022 |
|  | *p__Gemmatimonadota* | CK | 4.188 | 3.807 | 0.025 |
|  | *p__Acidobacteriota;c__Acidobacteriae;o__Solibacterales* | CK | 4.001 | 3.667 | 0.022 |
|  | *p__Actinobacteriota;c__Actinobacteria;o__Micrococcales* | SNF1 | 4.199 | 3.679 | 0.016 |
|  | *p__Proteobacteria;c__Gammaproteobacteria;o__KF-JG30-C25;f__norank_o__KF-JG30-C25* | SNF2 | 4.275 | 3.787 | 0.050 |
|  | *p__Chloroflexi;c__AD3* | SNF1 | 5.000 | 4.621 | 0.031 |
|  | *p__Acidobacteriota;c__Acidobacteriae;o__Acidobacteriales;f__Acidobacteriaceae_Subgroup_1;g__Occallatibacter* | CK | 4.259 | 3.869 | 0.038 |
|  | *p__Actinobacteriota;c__Actinobacteria* | CF | 5.420 | 4.789 | 0.038 |
|  | *p__Firmicutes;c__Bacilli;o__Paenibacillales;f__Paenibacillaceae;g__Paenibacillus* | CF | 4.168 | 3.768 | 0.024 |
|  | *p__Proteobacteria;c__Alphaproteobacteria;o__Rhizobiales;f__Xanthobacteraceae;g__norank_f__Xanthobacteraceae* | CK | 3.959 | 3.567 | 0.027 |
|  | *p__Acidobacteriota;c__Acidobacteriae* | CK | 5.190 | 4.764 | 0.038 |
|  | *p__Actinobacteriota;c__Actinobacteria;o__Corynebacteriales;f__Mycobacteriaceae;g__Mycobacterium* | CF | 4.169 | 3.676 | 0.050 |
|  | *p__Proteobacteria;c__Gammaproteobacteria;o__Burkholderiales;f__SC-I-84* | CK | 3.881 | 3.540 | 0.016 |
|  | *p__Gemmatimonadota;c__Gemmatimonadetes;o__Gemmatimonadales* | CK | 4.165 | 3.836 | 0.022 |
|  | *p__Acidobacteriota;c__Acidobacteriae;o__Acidobacteriales* | CK | 4.923 | 4.543 | 0.038 |
|  | *p__Proteobacteria;c__Alphaproteobacteria;o__Acetobacterales* | CF | 4.371 | 3.837 | 0.050 |
|  | *p__Acidobacteriota;c__Acidobacteriae;o__Solibacterales;f__Solibacteraceae;g__Candidatus_Solibacter* | CK | 4.001 | 3.682 | 0.022 |
|  | *p__Actinobacteriota;c__Actinobacteria;o__Frankiales* | CF | 5.283 | 4.706 | 0.038 |
|  | *p__Acidobacteriota;c__Acidobacteriae;o__Subgroup_2* | CK | 4.617 | 4.259 | 0.038 |
|  | *p__Acidobacteriota;c__unclassified_p__Acidobacteriota;o__unclassified_p__Acidobacteriota;f__unclassified_p__Acidobacteriota;g__unclassified_p__Acidobacteriota* | CF | 4.040 | 3.683 | 0.041 |
|  | *p__Firmicutes;c__Bacilli;o__Bacillales;f__Bacillaceae;g__Bacillus* | CF | 4.369 | 3.946 | 0.022 |
|  | *p__Proteobacteria;c__Alphaproteobacteria;o__Rhizobiales* | CK | 4.714 | 4.281 | 0.019 |
|  | *p__Acidobacteriota;c__unclassified_p__Acidobacteriota* | CF | 4.040 | 3.656 | 0.041 |
|  | *p__Chloroflexi;c__Chloroflexia* | CF | 4.633 | 4.167 | 0.027 |
|  | *p__Chloroflexi;c__AD3;o__norank_c__AD3;f__norank_o__norank_c__AD3;g__norank_f__norank_o__norank_c__AD3* | SNF1 | 5.000 | 4.613 | 0.031 |
|  | *p__Proteobacteria;c__Gammaproteobacteria;o__Xanthomonadales* | CF | 4.682 | 4.323 | 0.019 |
|  | *p__Firmicutes;c__Bacilli;o__Bacillales;f__Bacillaceae* | CF | 4.384 | 3.924 | 0.022 |
|  | *p__Firmicutes;c__Sulfobacillia* | CF | 3.832 | 3.545 | 0.016 |
|  | *p__Actinobacteriota;c__Actinobacteria;o__Catenulisporales* | SNF1 | 4.088 | 3.548 | 0.034 |
|  | *p__Acidobacteriota;c__Acidobacteriae;o__Acidobacteriales;f__norank_o__Acidobacteriales;g__norank_f__norank_o__Acidobacteriales* | CK | 4.651 | 4.293 | 0.022 |
|  | *p__GAL15;c__norank_p__GAL15;o__norank_c__norank_p__GAL15;f__norank_o__norank_c__norank_p__GAL15;g__norank_f__norank_o__norank_c__norank_p__GAL15* | CK | 4.006 | 3.689 | 0.018 |
|  | *p__Firmicutes;c__Bacilli;o__Paenibacillales* | CF | 4.350 | 3.901 | 0.019 |
|  | *p__Acidobacteriota;c__Acidobacteriae;o__Subgroup_2;f__norank_o__Subgroup_2* | CK | 4.617 | 4.294 | 0.038 |
|  | *p__Gemmatimonadota;c__Gemmatimonadetes;o__Gemmatimonadales;f__Gemmatimonadaceae;g__norank_f__Gemmatimonadaceae* | CK | 4.048 | 3.713 | 0.016 |
|  | *p__GAL15* | CK | 4.006 | 3.693 | 0.018 |
|  | *p__Proteobacteria;c__Alphaproteobacteria;o__Acetobacterales;f__Acetobacteraceae;g__unclassified_f__Acetobacteraceae* | CF | 4.253 | 3.748 | 0.024 |
|  | *p__Chloroflexi;c__TK10;o__norank_c__TK10* | SNF1 | 4.227 | 3.916 | 0.022 |
|  | *p__Acidobacteriota;c__unclassified_p__Acidobacteriota;o__unclassified_p__Acidobacteriota* | CF | 4.040 | 3.659 | 0.041 |
|  | *p__Proteobacteria;c__Alphaproteobacteria;o__Acetobacterales;f__Acetobacteraceae* | CF | 4.371 | 3.835 | 0.050 |
|  | *p__Chloroflexi;c__TK10;o__norank_c__TK10;f__norank_o__norank_c__TK10;g__norank_f__norank_o__norank_c__TK10* | SNF1 | 4.227 | 3.917 | 0.022 |
|  | *p__Chloroflexi;c__TK10;o__norank_c__TK10;f__norank_o__norank_c__TK10* | SNF1 | 4.227 | 3.882 | 0.022 |
|  | *p__Actinobacteriota;c__Actinobacteria;o__Corynebacteriales;f__Mycobacteriaceae* | CF | 4.169 | 3.662 | 0.050 |
|  | *p__GAL15;c__norank_p__GAL15;o__norank_c__norank_p__GAL15* | CK | 4.006 | 3.717 | 0.018 |
|  | *p__Proteobacteria;c__Alphaproteobacteria;o__Elsterales;f__norank_o__Elsterales* | CK | 4.759 | 4.077 | 0.050 |
|  | *p__Actinobacteriota;c__Actinobacteria;o__Frankiales;f__Geodermatophilaceae* | SNF1 | 3.921 | 3.522 | 0.025 |
|  | *p__Acidobacteriota;c__Acidobacteriae;o__Acidobacteriales;f__norank_o__Acidobacteriales* | CK | 4.651 | 4.301 | 0.022 |
|  | *p__Patescibacteria;c__Saccharimonadia;o__Saccharimonadales* | SNF1 | 4.015 | 3.530 | 0.041 |
|  | *p__Firmicutes* | CF | 4.898 | 4.463 | 0.024 |
|  | *p__Actinobacteriota;c__Actinobacteria;o__Frankiales;f__Frankiaceae* | CF | 4.386 | 4.032 | 0.022 |
|  | *p__Proteobacteria;c__Gammaproteobacteria;o__Xanthomonadales;f__Rhodanobacteraceae* | CF | 4.682 | 4.317 | 0.019 |
|  | *p__Chloroflexi;c__Chloroflexia;o__Thermomicrobiales;f__JG30-KF-CM45* | CF | 4.488 | 4.069 | 0.038 |
|  | *p__Proteobacteria;c__Alphaproteobacteria;o__Elsterales* | CK | 4.763 | 4.147 | 0.050 |
|  | *p__Chloroflexi;c__AD3;o__norank_c__AD3;f__norank_o__norank_c__AD3* | SNF1 | 5.000 | 4.605 | 0.031 |
|  | *p__Proteobacteria;c__Gammaproteobacteria;o__KF-JG30-C25;f__norank_o__KF-JG30-C25;g__norank_f__norank_o__KF-JG30-C25* | SNF2 | 4.275 | 3.815 | 0.050 |
|  | *p__Actinobacteriota;c__Actinobacteria;o__Frankiales;f__Acidothermaceae* | SNF2 | 5.202 | 4.577 | 0.038 |
|  | *p__Proteobacteria;c__Alphaproteobacteria;o__Sphingomonadales;f__Sphingomonadaceae;g__Sphingomonas* | SNF1 | 3.961 | 3.672 | 0.016 |
|  | *p__Proteobacteria;c__Gammaproteobacteria;o__Burkholderiales;f__SC-I-84;g__norank_f__SC-I-84* | CK | 3.881 | 3.533 | 0.016 |
|  | *p__Firmicutes;c__Sulfobacillia;o__Sulfobacillales* | CF | 3.832 | 3.513 | 0.016 |
|  | *p__Proteobacteria;c__Alphaproteobacteria;o__Elsterales;f__norank_o__Elsterales;g__norank_f__norank_o__Elsterales* | CK | 4.759 | 4.121 | 0.050 |
|  | *p__Actinobacteriota;c__Actinobacteria;o__Frankiales;f__Geodermatophilaceae;g__Geodermatophilus* | SNF2 | 3.909 | 3.508 | 0.025 |
|  | *p__Acidobacteriota;c__unclassified_p__Acidobacteriota;o__unclassified_p__Acidobacteriota;f__unclassified_p__Acidobacteriota;* | CF | 4.040 | 3.657 | 0.041 |
|  | *p__Chloroflexi;c__AD3;o__norank_c__AD3* | SNF1 | 5.000 | 4.632 | 0.031 |
|  | *p__Chloroflexi;c__Chloroflexia;o__Thermomicrobiales;f__JG30-KF-CM45;g__norank_f__JG30-KF-CM45* | CF | 4.488 | 4.060 | 0.038 |
|  | *p__Proteobacteria;c__Gammaproteobacteria;o__Burkholderiales* | CK | 4.103 | 3.725 | 0.029 |
|  | *p__Proteobacteria;c__Alphaproteobacteria;o__Rhizobiales;f__Xanthobacteraceae;g__Bradyrhizobium* | SNF1 | 4.175 | 3.679 | 0.026 |
|  | *p__Chloroflexi;c__Chloroflexia;o__Thermomicrobiales* | CF | 4.560 | 4.149 | 0.023 |
|  | *p__Proteobacteria;c__Alphaproteobacteria* | CK | 5.160 | 4.532 | 0.044 |
|  | *p__Firmicutes;c__Sulfobacillia;o__Sulfobacillales;f__Sulfobacillaceae;g__norank_f__Sulfobacillaceae* | CF | 3.832 | 3.531 | 0.016 |
|  | *p__Proteobacteria;c__Alphaproteobacteria;o__Sphingomonadales;f__Sphingomonadaceae* | SNF1 | 3.985 | 3.666 | 0.016 |
|  | *p__Firmicutes;c__Sulfobacillia;o__Sulfobacillales;f__Sulfobacillaceae* | CF | 3.832 | 3.543 | 0.016 |
|  | *p__Proteobacteria;c__Gammaproteobacteria;o__KF-JG30-C25* | SNF2 | 4.275 | 3.789 | 0.050 |
|  | *p__Actinobacteriota;c__Actinobacteria;o__Frankiales;f__Frankiaceae;g__Jatrophihabitans* | CF | 4.386 | 3.995 | 0.022 |
|  | *p__Firmicutes;c__Bacilli;o__Paenibacillales;f__Paenibacillaceae* | CF | 4.350 | 3.950 | 0.019 |
|  | *p__Actinobacteriota;c__Actinobacteria;o__Micrococcales;f__Intrasporangiaceae* | CF | 3.910 | 3.529 | 0.024 |
|  | *p__Proteobacteria;c__Alphaproteobacteria;o__Rhizobiales;f__Xanthobacteraceae* | CK | 4.585 | 4.141 | 0.019 |
|  | *p__Patescibacteria;c__Saccharimonadia* | SNF1 | 4.015 | 3.528 | 0.041 |
|  | *p__Proteobacteria;c__Gammaproteobacteria;o__Xanthomonadales;f__Rhodanobacteraceae;g__Chujaibacter* | CF | 4.673 | 4.373 | 0.016 |
|  | *p__Firmicutes;c__Bacilli;o__Bacillales;f__Planococcaceae* | CF | 4.019 | 3.579 | 0.025 |
|  | *p__Chloroflexi;c__TK10* | SNF1 | 4.227 | 3.928 | 0.022 |
|  | *p__Gemmatimonadota;c__Gemmatimonadetes* | CK | 4.165 | 3.823 | 0.022 |
|  | *p__Acidobacteriota;c__Acidobacteriae;o__Solibacterales;f__Solibacteraceae* | CK | 4.001 | 3.674 | 0.022 |
|  | *p__Patescibacteria* | SNF1 | 4.065 | 3.553 | 0.038 |
|  | *p__GAL15;c__norank_p__GAL15* | CK | 4.006 | 3.731 | 0.018 |
|  | *p__Firmicutes;c__Bacilli* | CF | 4.816 | 4.385 | 0.024 |
|  | *p__Proteobacteria;c__Alphaproteobacteria;o__Sphingomonadales* | SNF1 | 3.985 | 3.653 | 0.016 |
| Fungal | *p__Ascomycota;c__Sordariomycetes;o__Ophiostomatales;f__Ophiostomataceae* | SNF1 | 4.063 | 3.701 | 0.022 |
|  | *p__Basidiomycota;c__Agaricomycetes;o__Trechisporales* | SNF2 | 4.516 | 4.193 | 0.016 |
|  | *p__Mortierellomycota* | SNF2 | 4.915 | 4.556 | 0.016 |
|  | *p__Ascomycota;c__Leotiomycetes;o__Helotiales;f__Helotiales_fam_Incertae_sedis;g__Kendrickiella* | CF | 4.351 | 4.030 | 0.021 |
|  | *p__Ascomycota;c__Eurotiomycetes;o__Eurotiales;f__Trichocomaceae;g__Talaromyces* | CF | 5.113 | 4.627 | 0.034 |
|  | *p__Ascomycota;c__Sordariomycetes;o__Sordariales;f__Chaetomiaceae;g__Myceliophthora* | SNF2 | 3.948 | 3.603 | 0.027 |
|  | *p__unclassified_k__Fungi;c__unclassified_k__Fungi;o__unclassified_k__Fungi* | SNF1 | 4.244 | 3.857 | 0.025 |
|  | *p__Ascomycota;c__Sordariomycetes;o__Diaporthales;f__Melanconidaceae* | CK | 4.236 | 3.878 | 0.016 |
|  | *p__Ascomycota;c__Sordariomycetes;o__Hypocreales;f__Hypocreaceae* | SNF1 | 5.038 | 4.349 | 0.038 |
|  | *p__Basidiomycota;c__Agaricomycetes;o__Agaricales;f__unclassified_o__Agaricales;g__unclassified_o__Agaricales* | SNF1 | 3.863 | 3.532 | 0.015 |
|  | *p__Basidiomycota;c__Tremellomycetes;o__Tremellales;f__unclassified_o__Tremellales;g__unclassified_o__Tremellales* | CF | 4.598 | 4.312 | 0.024 |
|  | *p__Ascomycota;c__Eurotiomycetes;o__Eurotiales;f__Aspergillaceae* | SNF2 | 4.967 | 4.462 | 0.038 |
|  | *p__Ascomycota;c__unclassified_p__Ascomycota;o__unclassified_p__Ascomycota* | SNF2 | 4.472 | 3.954 | 0.024 |
|  | *p__Mortierellomycota;c__Mortierellomycetes;o__Mortierellales* | SNF2 | 4.915 | 4.541 | 0.016 |
|  | *p__Basidiomycota;c__Agaricomycetes;o__Phallales* | SNF1 | 4.568 | 4.263 | 0.042 |
|  | *p__Basidiomycota;c__Agaricomycetes;o__Trechisporales;f__Hydnodontaceae* | SNF2 | 4.515 | 4.177 | 0.016 |
|  | *p__Ascomycota;c__Eurotiomycetes;o__Chaetothyriales;f__unclassified_o__Chaetothyriales;g__unclassified_o__Chaetothyriales* | CF | 4.487 | 3.981 | 0.024 |
|  | *p__Ascomycota;c__Leotiomycetes;o__Helotiales;f__Helotiales_fam_Incertae_sedis* | CF | 4.360 | 4.033 | 0.038 |
|  | *p__Ascomycota;c__Eurotiomycetes;o__Chaetothyriales* | SNF2 | 4.795 | 4.285 | 0.033 |
|  | *p__Basidiomycota;c__Agaricomycetes;o__Agaricales* | SNF1 | 4.266 | 3.964 | 0.016 |
|  | *p__Basidiomycota;c__Agaricomycetes;o__Phallales;f__Phallaceae* | SNF1 | 4.568 | 4.288 | 0.042 |
|  | *p__Ascomycota;c__unclassified_p__Ascomycota;o__unclassified_p__Ascomycota;f__unclassified_p__Ascomycota* | SNF2 | 4.472 | 4.004 | 0.024 |
|  | *p__Mortierellomycota;c__Mortierellomycetes;o__Mortierellales;f__Mortierellaceae* | SNF2 | 4.915 | 4.548 | 0.016 |
|  | *p__Basidiomycota;c__Agaricomycetes;o__Agaricales;f__Agaricaceae* | SNF1 | 3.999 | 3.727 | 0.031 |
|  | *p__Ascomycota;c__Eurotiomycetes;o__Eurotiales;f__Aspergillaceae;g__Penicillium* | SNF2 | 4.937 | 4.443 | 0.041 |
|  | *p__Kickxellomycota;c__Kickxellomycetes;o__Kickxellales;f__Kickxellaceae;g__Ramicandelaber* | CK | 2.343 | 3.524 | 0.017 |
|  | *p__Basidiomycota;c__Agaricomycetes;o__Agaricales;f__unclassified_o__Agaricales* | SNF1 | 3.863 | 3.541 | 0.015 |
|  | *p__Basidiomycota;c__Tremellomycetes;o__Tremellales;f__Rhynchogastremataceae* | SNF1 | 4.143 | 3.775 | 0.033 |
|  | *p__Basidiomycota;c__Agaricomycetes;o__Phallales;f__Phallaceae;g__Phallus* | SNF1 | 4.568 | 4.271 | 0.042 |
|  | *p__Mortierellomycota;c__Mortierellomycetes* | SNF2 | 4.915 | 4.560 | 0.016 |
|  | *p__Ascomycota;c__Leotiomycetes* | CF | 4.380 | 4.060 | 0.041 |
|  | *p__Ascomycota;c__Eurotiomycetes;o__Chaetothyriales;f__unclassified_o__Chaetothyriales* | CF | 4.487 | 3.997 | 0.024 |
|  | *p__unclassified_k__Fungi;c__unclassified_k__Fungi;o__unclassified_k__Fungi;f__unclassified_k__Fungi* | SNF1 | 4.244 | 3.837 | 0.025 |
|  | *p__Ascomycota;c__Eurotiomycetes;o__Chaetothyriales;f__Herpotrichiellaceae;g__Coniosporium* | SNF2 | 4.263 | 3.884 | 0.033 |
|  | *p__Basidiomycota;c__Agaricomycetes* | SNF1 | 4.802 | 4.458 | 0.016 |
|  | *p__Ascomycota;c__Sordariomycetes;o__unclassified_c__Sordariomycetes;f__unclassified_c__Sordariomycetes;g__unclassified_c__Sordariomycetes* | CK | 4.332 | 3.858 | 0.024 |
|  | *p__Ascomycota;c__Eurotiomycetes;o__Eurotiales;f__Trichocomaceae* | CF | 5.115 | 4.593 | 0.034 |
|  | *p__Mortierellomycota;c__Mortierellomycetes;o__Mortierellales;f__Mortierellaceae;g__Mortierella* | SNF2 | 4.915 | 4.550 | 0.016 |
|  | *p__unclassified_k__Fungi* | SNF1 | 4.244 | 3.848 | 0.025 |
|  | *p__Ascomycota;c__unclassified_p__Ascomycota;o__unclassified_p__Ascomycota;f__unclassified_p__Ascomycota;g__unclassified_p__Ascomycota* | SNF2 | 4.472 | 3.993 | 0.024 |
|  | *p__unclassified_k__Fungi;c__unclassified_k__Fungi* | SNF1 | 4.244 | 3.839 | 0.025 |
|  | *p__Basidiomycota;c__Tremellomycetes;o__Tremellales;f__Rhynchogastremataceae;g__Papiliotrema* | SNF1 | 4.143 | 3.785 | 0.033 |
|  | *p__Ascomycota;c__Sordariomycetes;o__Hypocreales;f__Hypocreaceae;g__Trichoderma* | SNF1 | 5.036 | 4.347 | 0.038 |
|  | *p__Ascomycota;c__unclassified_p__Ascomycota* | SNF2 | 4.472 | 3.996 | 0.024 |
|  | *p__Ascomycota;c__Dothideomycetes* | SNF2 | 3.958 | 3.549 | 0.019 |
|  | *p__Ascomycota;c__Leotiomycetes;o__Helotiales* | CF | 4.378 | 4.056 | 0.041 |
|  | *p__Ascomycota;c__Sordariomycetes;o__Ophiostomatales* | SNF1 | 4.063 | 3.693 | 0.022 |
|  | *p__Ascomycota;c__Dothideomycetes;o__Pleosporales;f__Didymosphaeriaceae;g__Pseudopithomyces* | SNF1 | 1.794 | 3.812 | 0.041 |
|  | *p__Basidiomycota;c__Agaricomycetes;o__Trechisporales;f__Hydnodontaceae;g__Trechispora* | SNF2 | 4.515 | 4.209 | 0.016 |
|  | *p__unclassified_k__Fungi;c__unclassified_k__Fungi;o__unclassified_k__Fungi;f__unclassified_k__Fungi;g__unclassified_k__Fungi* | SNF1 | 4.244 | 3.861 | 0.025 |
|  | *p__Ascomycota;c__Sordariomycetes;o__Hypocreales* | SNF1 | 5.180 | 4.289 | 0.038 |
|  | *p__Ascomycota;c__Sordariomycetes;o__Diaporthales;f__Melanconidaceae;g__Melanconiella* | CK | 4.236 | 3.905 | 0.016 |
|  | *p__Basidiomycota;c__Tremellomycetes;o__Tremellales;f__unclassified_o__Tremellales* | CF | 4.598 | 4.288 | 0.024 |
|  | *p__Ascomycota;c__Sordariomycetes;o__unclassified_c__Sordariomycetes* | CK | 4.332 | 3.877 | 0.024 |
|  | *p__Ascomycota;c__Sordariomycetes;o__unclassified_c__Sordariomycetes;f__unclassified_c__Sordariomycetes;* | CK | 4.332 | 3.889 | 0.024 |
|  | *p__Ascomycota;c__Sordariomycetes;o__Diaporthales* | CK | 4.236 | 3.883 | 0.016 |
|  | *p__Ascomycota;c__Sordariomycetes;o__Ophiostomatales;f__Ophiostomataceae;g__Sporothrix* | SNF1 | 3.969 | 3.607 | 0.025 |
|  | *p__Basidiomycota;c__Agaricomycetes;o__Agaricales;f__Psathyrellaceae;g__Coprinellus* | SNF2 | 1.898 | 3.568 | 0.042 |

CK, CF, SNF1 and SNF2 stand for non fertilizer, conventional fertilizer, 15% less than CF and 20% less than CF. LDA score > 3.5 (*P* < 0.05, n = 3).

**Supplementary Table 4.** The two-factor correlation network of bacterial and fungal communities with soil factors in 2020

| Species name | | Soil factors | Correlation | *P*_value |
| --- | --- | --- | --- | --- |
| Bacterial | *p__Acidobacteriota; c__Acidobacteriae; o__Acidobacteriales; f__Acidobacteriaceae_Subgroup_1; g__Occallatibacter* | pH | 0.832 | 0.001 |
|  | *p__Acidobacteriota; c__Acidobacteriae; o__Acidobacteriales; f__Acidobacteriaceae_Subgroup_1; g__Occallatibacter* | EC | -0.887 | 0.000 |
|  | *p__Acidobacteriota; c__Acidobacteriae; o__Acidobacteriales; f__Acidobacteriaceae_Subgroup_1; g__Occallatibacter* | Nmin | -0.811 | 0.001 |
|  | *p__Acidobacteriota; c__Acidobacteriae; o__Acidobacteriales; f__Acidobacteriaceae_Subgroup_1; g__Occallatibacter* | K^+^ | -0.718 | 0.009 |
|  | *p__Acidobacteriota; c__Acidobacteriae; o__Acidobacteriales; f__Acidobacteriaceae_Subgroup_1; g__Occallatibacter* | Ca^2+^ | -0.594 | 0.042 |
|  | *p__Acidobacteriota; c__Acidobacteriae; o__Acidobacteriales; f__Acidobacteriaceae_Subgroup_1; g__Occallatibacter* | SOM | 0.685 | 0.014 |
|  | *p__Acidobacteriota; c__Acidobacteriae; o__Acidobacteriales; f__Acidobacteriaceae_Subgroup_1; g__Occallatibacter* | urease | 0.734 | 0.007 |
|  | *p__Acidobacteriota; c__Acidobacteriae; o__Acidobacteriales; f__Acidobacteriaceae_Subgroup_1; g__unclassified_f__Acidobacteriaceae_Subgroup_1* | pH | 0.727 | 0.007 |
|  | *p__Acidobacteriota; c__Acidobacteriae; o__Acidobacteriales; f__Acidobacteriaceae_Subgroup_1; g__unclassified_f__Acidobacteriaceae_Subgroup_1* | EC | -0.813 | 0.001 |
|  | *p__Acidobacteriota; c__Acidobacteriae; o__Acidobacteriales; f__Acidobacteriaceae_Subgroup_1; g__unclassified_f__Acidobacteriaceae_Subgroup_1* | Nmin | -0.741 | 0.006 |
|  | *p__Acidobacteriota; c__Acidobacteriae; o__Acidobacteriales; f__Acidobacteriaceae_Subgroup_1; g__unclassified_f__Acidobacteriaceae_Subgroup_1* | K^+^ | -0.687 | 0.014 |
|  | *p__Acidobacteriota; c__Acidobacteriae; o__Acidobacteriales; f__Acidobacteriaceae_Subgroup_1; g__unclassified_f__Acidobacteriaceae_Subgroup_1* | SOM | 0.755 | 0.005 |
|  | *p__Acidobacteriota; c__Acidobacteriae; o__Acidobacteriales; f__Acidobacteriaceae_Subgroup_1; g__unclassified_f__Acidobacteriaceae_Subgroup_1* | urease | 0.713 | 0.009 |
|  | *p__Acidobacteriota; c__Acidobacteriae; o__Acidobacteriales; f__norank_o__Acidobacteriales; g__norank_f__norank_o__Acidobacteriales* | pH | 0.874 | 0.000 |
|  | *p__Acidobacteriota; c__Acidobacteriae; o__Acidobacteriales; f__norank_o__Acidobacteriales; g__norank_f__norank_o__Acidobacteriales* | EC | -0.940 | 0.000 |
|  | *p__Acidobacteriota; c__Acidobacteriae; o__Acidobacteriales; f__norank_o__Acidobacteriales; g__norank_f__norank_o__Acidobacteriales* | Nmin | -0.888 | 0.000 |
|  | *p__Acidobacteriota; c__Acidobacteriae; o__Acidobacteriales; f__norank_o__Acidobacteriales; g__norank_f__norank_o__Acidobacteriales* | K^+^ | -0.694 | 0.012 |
|  | *p__Acidobacteriota; c__Acidobacteriae; o__Acidobacteriales; f__norank_o__Acidobacteriales; g__norank_f__norank_o__Acidobacteriales* | SOM | 0.748 | 0.005 |
|  | *p__Acidobacteriota; c__Acidobacteriae; o__Acidobacteriales; f__norank_o__Acidobacteriales; g__norank_f__norank_o__Acidobacteriales* | urease | 0.818 | 0.001 |
|  | *p__Acidobacteriota; c__Acidobacteriae; o__Bryobacterales; f__Bryobacteraceae; g__Bryobacter* | K^+^ | 0.609 | 0.035 |
|  | *p__Acidobacteriota; c__Acidobacteriae; o__Bryobacterales; f__Bryobacteraceae; g__Bryobacter* | Ca^2+^ | 0.685 | 0.014 |
|  | *p__Acidobacteriota; c__Acidobacteriae; o__Solibacterales; f__Solibacteraceae; g__Candidatus_Solibacter* | pH | 0.867 | 0.000 |
|  | *p__Acidobacteriota; c__Acidobacteriae; o__Solibacterales; f__Solibacteraceae; g__Candidatus_Solibacter* | EC | -0.908 | 0.000 |
|  | *p__Acidobacteriota; c__Acidobacteriae; o__Solibacterales; f__Solibacteraceae; g__Candidatus_Solibacter* | Nmin | -0.895 | 0.000 |
|  | *p__Acidobacteriota; c__Acidobacteriae; o__Solibacterales; f__Solibacteraceae; g__Candidatus_Solibacter* | K^+^ | -0.690 | 0.013 |
|  | *p__Acidobacteriota; c__Acidobacteriae; o__Solibacterales; f__Solibacteraceae; g__Candidatus_Solibacter* | SOM | 0.685 | 0.014 |
|  | *p__Acidobacteriota; c__Acidobacteriae; o__Solibacterales; f__Solibacteraceae; g__Candidatus_Solibacter* | urease | 0.832 | 0.001 |
|  | *p__Acidobacteriota; c__Acidobacteriae; o__Solibacterales; f__Solibacteraceae; g__Candidatus_Solibacter* | acid phosphata-se | 0.615 | 0.033 |
|  | *p__Acidobacteriota; c__Acidobacteriae; o__Subgroup_2; f__norank_o__Subgroup_2; g__norank_f__norank_o__Subgroup_2* | pH | 0.823 | 0.001 |
|  | *p__Acidobacteriota; c__Acidobacteriae; o__Subgroup_2; f__norank_o__Subgroup_2; g__norank_f__norank_o__Subgroup_2* | EC | -0.899 | 0.000 |
|  | *p__Acidobacteriota; c__Acidobacteriae; o__Subgroup_2; f__norank_o__Subgroup_2; g__norank_f__norank_o__Subgroup_2* | Nmin | -0.823 | 0.001 |
|  | *p__Acidobacteriota; c__Acidobacteriae; o__Subgroup_2; f__norank_o__Subgroup_2; g__norank_f__norank_o__Subgroup_2* | K^+^ | -0.726 | 0.007 |
|  | *p__Acidobacteriota; c__Acidobacteriae; o__Subgroup_2; f__norank_o__Subgroup_2; g__norank_f__norank_o__Subgroup_2* | Ca^2+^ | -0.602 | 0.038 |
|  | *p__Acidobacteriota; c__Acidobacteriae; o__Subgroup_2; f__norank_o__Subgroup_2; g__norank_f__norank_o__Subgroup_2* | SOM | 0.711 | 0.010 |
|  | *p__Acidobacteriota; c__Acidobacteriae; o__Subgroup_2; f__norank_o__Subgroup_2; g__norank_f__norank_o__Subgroup_2* | urease | 0.760 | 0.004 |
|  | *p__Acidobacteriota; c__unclassified_p__Acidobacteriota; o__unclassified_p__Acidobacteriota; f__unclassified_p__Acidobacteriota; g__unclassified_p__Acidobacteriota* | pH | -0.727 | 0.007 |
|  | *p__Acidobacteriota; c__unclassified_p__Acidobacteriota; o__unclassified_p__Acidobacteriota; f__unclassified_p__Acidobacteriota; g__unclassified_p__Acidobacteriota* | EC | 0.771 | 0.003 |
|  | *p__Acidobacteriota; c__unclassified_p__Acidobacteriota; o__unclassified_p__Acidobacteriota; f__unclassified_p__Acidobacteriota; g__unclassified_p__Acidobacteriota* | Nmin | 0.825 | 0.001 |
|  | *p__Acidobacteriota; c__unclassified_p__Acidobacteriota; o__unclassified_p__Acidobacteriota; f__unclassified_p__Acidobacteriota; g__unclassified_p__Acidobacteriota* | SOM | -0.657 | 0.020 |
|  | *p__Acidobacteriota; c__unclassified_p__Acidobacteriota; o__unclassified_p__Acidobacteriota; f__unclassified_p__Acidobacteriota; g__unclassified_p__Acidobacteriota* | urease | -0.797 | 0.002 |
|  | *p__Acidobacteriota; c__unclassified_p__Acidobacteriota; o__unclassified_p__Acidobacteriota; f__unclassified_p__Acidobacteriota; g__unclassified_p__Acidobacteriota* | acid phosphata-se | -0.615 | 0.033 |
|  | *p__Actinobacteriota; c__Acidimicrobiia; o__IMCC26256; f__norank_o__IMCC26256; g__norank_f__norank_o__IMCC26256* | AP | 0.599 | 0.040 |
|  | *p__Actinobacteriota; c__Acidimicrobiia; o__IMCC26256; f__norank_o__IMCC26256; g__norank_f__norank_o__IMCC26256* | Mg^2+^ | -0.683 | 0.014 |
|  | *p__Actinobacteriota; c__Acidimicrobiia; o__norank_c__Acidimicrobiia; f__norank_o__norank_c__Acidimicrobiia; g__norank_f__norank_o__norank_c__Acidimicrobiia* | EC | -0.623 | 0.030 |
|  | *p__Actinobacteriota; c__Acidimicrobiia; o__norank_c__Acidimicrobiia; f__norank_o__norank_c__Acidimicrobiia; g__norank_f__norank_o__norank_c__Acidimicrobiia* | Nmin | -0.615 | 0.033 |
|  | *p__Actinobacteriota; c__Actinobacteria; o__Catenulisporales; f__Actinospicaceae; g__Actinospica* | AP | 0.666 | 0.018 |
|  | *p__Actinobacteriota; c__Actinobacteria; o__Corynebacteriales; f__Mycobacteriaceae; g__Mycobacterium* | pH | -0.839 | 0.001 |
|  | *p__Actinobacteriota; c__Actinobacteria; o__Corynebacteriales; f__Mycobacteriaceae; g__Mycobacterium* | EC | 0.813 | 0.001 |
|  | *p__Actinobacteriota; c__Actinobacteria; o__Corynebacteriales; f__Mycobacteriaceae; g__Mycobacterium* | Nmin | 0.790 | 0.002 |
|  | *p__Actinobacteriota; c__Actinobacteria; o__Corynebacteriales; f__Mycobacteriaceae; g__Mycobacterium* | K^+^ | 0.736 | 0.006 |
|  | *p__Actinobacteriota; c__Actinobacteria; o__Corynebacteriales; f__Mycobacteriaceae; g__Mycobacterium* | Ca^2+^ | 0.615 | 0.033 |
|  | *p__Actinobacteriota; c__Actinobacteria; o__Corynebacteriales; f__Mycobacteriaceae; g__Mycobacterium* | SOM | -0.594 | 0.042 |
|  | *p__Actinobacteriota; c__Actinobacteria; o__Corynebacteriales; f__Mycobacteriaceae; g__Mycobacterium* | urease | -0.741 | 0.006 |
|  | *p__Actinobacteriota; c__Actinobacteria; o__Frankiales; f__Acidothermaceae; g__Acidothermus* | pH | -0.846 | 0.001 |
|  | *p__Actinobacteriota; c__Actinobacteria; o__Frankiales; f__Acidothermaceae; g__Acidothermus* | EC | 0.866 | 0.000 |
|  | *p__Actinobacteriota; c__Actinobacteria; o__Frankiales; f__Acidothermaceae; g__Acidothermus* | Nmin | 0.825 | 0.001 |
|  | *p__Actinobacteriota; c__Actinobacteria; o__Frankiales; f__Acidothermaceae; g__Acidothermus* | K^+^ | 0.694 | 0.012 |
|  | *p__Actinobacteriota; c__Actinobacteria; o__Frankiales; f__Acidothermaceae; g__Acidothermus* | SOM | -0.643 | 0.024 |
|  | *p__Actinobacteriota; c__Actinobacteria; o__Frankiales; f__Acidothermaceae; g__Acidothermus* | urease | -0.811 | 0.001 |
|  | *p__Actinobacteriota; c__Actinobacteria; o__Frankiales; f__Frankiaceae; g__Jatrophihabitans* | pH | -0.914 | 0.000 |
|  | *p__Actinobacteriota; c__Actinobacteria; o__Frankiales; f__Frankiaceae; g__Jatrophihabitans* | EC | 0.952 | 0.000 |
|  | *p__Actinobacteriota; c__Actinobacteria; o__Frankiales; f__Frankiaceae; g__Jatrophihabitans* | Nmin | 0.900 | 0.000 |
|  | *p__Actinobacteriota; c__Actinobacteria; o__Frankiales; f__Frankiaceae; g__Jatrophihabitans* | K^+^ | 0.800 | 0.002 |
|  | *p__Actinobacteriota; c__Actinobacteria; o__Frankiales; f__Frankiaceae; g__Jatrophihabitans* | Ca^2+^ | 0.704 | 0.011 |
|  | *p__Actinobacteriota; c__Actinobacteria; o__Frankiales; f__Frankiaceae; g__Jatrophihabitans* | urease | -0.834 | 0.001 |
|  | *p__Actinobacteriota; c__Actinobacteria; o__Frankiales; f__Geodermatophilaceae; g__Geodermatophilus* | AP | 0.678 | 0.015 |
|  | *p__Actinobacteriota; c__Actinobacteria; o__Micromonosporales; f__Micromonosporaceae; g__unclassified_f__Micromonosporaceae* | pH | -0.636 | 0.026 |
|  | *p__Actinobacteriota; c__Actinobacteria; o__Micromonosporales; f__Micromonosporaceae; g__unclassified_f__Micromonosporaceae* | EC | 0.644 | 0.024 |
|  | *p__Actinobacteriota; c__Actinobacteria; o__Micromonosporales; f__Micromonosporaceae; g__unclassified_f__Micromonosporaceae* | Nmin | 0.636 | 0.026 |
|  | *p__Actinobacteriota; c__Actinobacteria; o__Micromonosporales; f__Micromonosporaceae; g__unclassified_f__Micromonosporaceae* | K^+^ | 0.753 | 0.005 |
|  | *p__Actinobacteriota; c__Actinobacteria; o__Micromonosporales; f__Micromonosporaceae; g__unclassified_f__Micromonosporaceae* | Ca^2+^ | 0.720 | 0.008 |
|  | *p__Actinobacteriota; c__Actinobacteria; o__Streptomycetales; f__Streptomycetaceae; g__Kitasatospora* | pH | 0.768 | 0.004 |
|  | *p__Actinobacteriota; c__Actinobacteria; o__Streptomycetales; f__Streptomycetaceae; g__Kitasatospora* | EC | -0.800 | 0.002 |
|  | *p__Actinobacteriota; c__Actinobacteria; o__Streptomycetales; f__Streptomycetaceae; g__Kitasatospora* | Nmin | -0.821 | 0.001 |
|  | *p__Actinobacteriota; c__Actinobacteria; o__Streptomycetales; f__Streptomycetaceae; g__Kitasatospora* | urease | 0.677 | 0.016 |
|  | *p__Actinobacteriota; c__Actinobacteria; o__Streptomycetales; f__Streptomycetaceae; g__Kitasatospora* | acid phosphata-se | 0.667 | 0.018 |
|  | *p__Actinobacteriota; c__Actinobacteria; o__Streptomycetales; f__Streptomycetaceae; g__Streptomyces* | pH | -0.622 | 0.031 |
|  | *p__Actinobacteriota; c__Actinobacteria; o__Streptomycetales; f__Streptomycetaceae; g__Streptomyces* | EC | 0.676 | 0.016 |
|  | *p__Actinobacteriota; c__Actinobacteria; o__Streptomycetales; f__Streptomycetaceae; g__Streptomyces* | Nmin | 0.594 | 0.042 |
|  | *p__Actinobacteriota; c__Actinobacteria; o__Streptomycetales; f__Streptomycetaceae; g__Streptomyces* | urease | -0.594 | 0.042 |
|  | *p__Actinobacteriota; c__Thermoleophilia; o__Gaiellales; f__norank_o__Gaiellales; g__norank_f__norank_o__Gaiellales* | pH | -0.615 | 0.033 |
|  | *p__Actinobacteriota; c__Thermoleophilia; o__Gaiellales; f__norank_o__Gaiellales; g__norank_f__norank_o__Gaiellales* | EC | 0.676 | 0.016 |
|  | *p__Actinobacteriota; c__Thermoleophilia; o__Gaiellales; f__norank_o__Gaiellales; g__norank_f__norank_o__Gaiellales* | urease | -0.643 | 0.024 |
|  | *p__Actinobacteriota; c__Thermoleophilia; o__Solirubrobacterales; f__Solirubrobacteraceae; g__Conexibacter* | pH | -0.587 | 0.045 |
|  | *p__Actinobacteriota; c__Thermoleophilia; o__Solirubrobacterales; f__Solirubrobacteraceae; g__Conexibacter* | EC | 0.708 | 0.010 |
|  | *p__Actinobacteriota; c__Thermoleophilia; o__Solirubrobacterales; f__Solirubrobacteraceae; g__Conexibacter* | Nmin | 0.643 | 0.024 |
|  | *p__Actinobacteriota; c__Thermoleophilia; o__Solirubrobacterales; f__Solirubrobacteraceae; g__Conexibacter* | SOM | -0.748 | 0.005 |
|  | *p__Actinobacteriota; c__Thermoleophilia; o__Solirubrobacterales; f__Solirubrobacteraceae; g__Conexibacter* | urease | -0.657 | 0.020 |
|  | *p__Chloroflexi; c__AD3; o__norank_c__AD3; f__norank_o__norank_c__AD3; g__norank_f__norank_o__norank_c__AD3* | pH | 0.804 | 0.002 |
|  | *p__Chloroflexi; c__AD3; o__norank_c__AD3; f__norank_o__norank_c__AD3; g__norank_f__norank_o__norank_c__AD3* | EC | -0.908 | 0.000 |
|  | *p__Chloroflexi; c__AD3; o__norank_c__AD3; f__norank_o__norank_c__AD3; g__norank_f__norank_o__norank_c__AD3* | Nmin | -0.818 | 0.001 |
|  | *p__Chloroflexi; c__AD3; o__norank_c__AD3; f__norank_o__norank_c__AD3; g__norank_f__norank_o__norank_c__AD3* | K^+^ | -0.655 | 0.021 |
|  | *p__Chloroflexi; c__AD3; o__norank_c__AD3; f__norank_o__norank_c__AD3; g__norank_f__norank_o__norank_c__AD3* | SOM | 0.594 | 0.042 |
|  | *p__Chloroflexi; c__AD3; o__norank_c__AD3; f__norank_o__norank_c__AD3; g__norank_f__norank_o__norank_c__AD3* | urease | 0.790 | 0.002 |
|  | *p__Chloroflexi; c__Chloroflexia; o__Thermomicrobiales; f__JG30-KF-CM45; g__norank_f__JG30-KF-CM45* | pH | -0.811 | 0.001 |
|  | *p__Chloroflexi; c__Chloroflexia; o__Thermomicrobiales; f__JG30-KF-CM45; g__norank_f__JG30-KF-CM45* | EC | 0.856 | 0.000 |
|  | *p__Chloroflexi; c__Chloroflexia; o__Thermomicrobiales; f__JG30-KF-CM45; g__norank_f__JG30-KF-CM45* | Nmin | 0.797 | 0.002 |
|  | *p__Chloroflexi; c__Chloroflexia; o__Thermomicrobiales; f__JG30-KF-CM45; g__norank_f__JG30-KF-CM45* | K^+^ | 0.760 | 0.004 |
|  | *p__Chloroflexi; c__Chloroflexia; o__Thermomicrobiales; f__JG30-KF-CM45; g__norank_f__JG30-KF-CM45* | urease | -0.846 | 0.001 |
|  | *p__Chloroflexi; c__JG30-KF-CM66; o__norank_c__JG30-KF-CM66; f__norank_o__norank_c__JG30-KF-CM66; g__norank_f__norank_o__norank_c__JG30-KF-CM66* | K^+^ | -0.725 | 0.008 |
|  | *p__Chloroflexi; c__JG30-KF-CM66; o__norank_c__JG30-KF-CM66; f__norank_o__norank_c__JG30-KF-CM66; g__norank_f__norank_o__norank_c__JG30-KF-CM66* | Ca^2+^ | -0.811 | 0.001 |
|  | *p__Chloroflexi; c__JG30-KF-CM66; o__norank_c__JG30-KF-CM66; f__norank_o__norank_c__JG30-KF-CM66; g__norank_f__norank_o__norank_c__JG30-KF-CM66* | sucrase | -0.782 | 0.003 |
|  | *p__Chloroflexi; c__Ktedonobacteria; o__B10-SB3A; f__norank_o__B10-SB3A; g__norank_f__norank_o__B10-SB3A* | EC | 0.634 | 0.027 |
|  | *p__Chloroflexi; c__Ktedonobacteria; o__B10-SB3A; f__norank_o__B10-SB3A; g__norank_f__norank_o__B10-SB3A* | Nmin | 0.622 | 0.031 |
|  | *p__Chloroflexi; c__Ktedonobacteria; o__B10-SB3A; f__norank_o__B10-SB3A; g__norank_f__norank_o__B10-SB3A* | SOM | -0.601 | 0.039 |
|  | *p__Chloroflexi; c__Ktedonobacteria; o__Ktedonobacterales; f__JG30-KF-AS9; g__norank_f__JG30-KF-AS9* | pH | -0.650 | 0.022 |
|  | *p__Chloroflexi; c__Ktedonobacteria; o__Ktedonobacterales; f__JG30-KF-AS9; g__norank_f__JG30-KF-AS9* | EC | 0.666 | 0.018 |
|  | *p__Chloroflexi; c__Ktedonobacteria; o__Ktedonobacterales; f__JG30-KF-AS9; g__norank_f__JG30-KF-AS9* | Nmin | 0.699 | 0.011 |
|  | *p__Chloroflexi; c__Ktedonobacteria; o__Ktedonobacterales; f__JG30-KF-AS9; g__norank_f__JG30-KF-AS9* | urease | -0.622 | 0.031 |
|  | *p__Chloroflexi; c__Ktedonobacteria; o__Ktedonobacterales; f__Ktedonobacteraceae; g__FCPS473* | pH | 0.580 | 0.048 |
|  | *p__Chloroflexi; c__Ktedonobacteria; o__Ktedonobacterales; f__Ktedonobacteraceae; g__FCPS473* | EC | -0.613 | 0.034 |
|  | *p__Chloroflexi; c__Ktedonobacteria; o__Ktedonobacterales; f__Ktedonobacteraceae; g__HSB_OF53-F07* | pH | 0.837 | 0.001 |
|  | *p__Chloroflexi; c__Ktedonobacteria; o__Ktedonobacterales; f__Ktedonobacteraceae; g__HSB_OF53-F07* | EC | -0.873 | 0.000 |
|  | *p__Chloroflexi; c__Ktedonobacteria; o__Ktedonobacterales; f__Ktedonobacteraceae; g__HSB_OF53-F07* | Nmin | -0.823 | 0.001 |
|  | *p__Chloroflexi; c__Ktedonobacteria; o__Ktedonobacterales; f__Ktedonobacteraceae; g__HSB_OF53-F07* | K^+^ | -0.737 | 0.006 |
|  | *p__Chloroflexi; c__Ktedonobacteria; o__Ktedonobacterales; f__Ktedonobacteraceae; g__HSB_OF53-F07* | SOM | 0.616 | 0.033 |
|  | *p__Chloroflexi; c__Ktedonobacteria; o__Ktedonobacterales; f__Ktedonobacteraceae; g__HSB_OF53-F07* | urease | 0.827 | 0.001 |
|  | *p__Chloroflexi; c__Ktedonobacteria; o__Ktedonobacterales; f__Ktedonobacteraceae; g__norank_f__Ktedonobacteraceae* | pH | -0.622 | 0.031 |
|  | *p__Chloroflexi; c__Ktedonobacteria; o__Ktedonobacterales; f__Ktedonobacteraceae; g__norank_f__Ktedonobacteraceae* | Nmin | 0.615 | 0.033 |
|  | *p__Chloroflexi; c__Ktedonobacteria; o__Ktedonobacterales; f__Ktedonobacteraceae; g__norank_f__Ktedonobacteraceae* | Ca^2+^ | 0.608 | 0.036 |
|  | *p__Chloroflexi; c__TK10; o__norank_c__TK10; f__norank_o__norank_c__TK10; g__norank_f__norank_o__norank_c__TK10* | pH | 0.774 | 0.003 |
|  | *p__Chloroflexi; c__TK10; o__norank_c__TK10; f__norank_o__norank_c__TK10; g__norank_f__norank_o__norank_c__TK10* | EC | -0.831 | 0.001 |
|  | *p__Chloroflexi; c__TK10; o__norank_c__TK10; f__norank_o__norank_c__TK10; g__norank_f__norank_o__norank_c__TK10* | Nmin | -0.750 | 0.005 |
|  | *p__Chloroflexi; c__TK10; o__norank_c__TK10; f__norank_o__norank_c__TK10; g__norank_f__norank_o__norank_c__TK10* | urease | 0.722 | 0.008 |
|  | *p__Chloroflexi; c__TK10; o__norank_c__TK10; f__norank_o__norank_c__TK10; g__norank_f__norank_o__norank_c__TK10* | acid phosphata-se | 0.722 | 0.008 |
|  | *p__Firmicutes; c__Bacilli; o__Bacillales; f__Bacillaceae; g__Bacillus* | pH | -0.825 | 0.001 |
|  | *p__Firmicutes; c__Bacilli; o__Bacillales; f__Bacillaceae; g__Bacillus* | EC | 0.792 | 0.002 |
|  | *p__Firmicutes; c__Bacilli; o__Bacillales; f__Bacillaceae; g__Bacillus* | Nmin | 0.762 | 0.004 |
|  | *p__Firmicutes; c__Bacilli; o__Bacillales; f__Bacillaceae; g__Bacillus* | K^+^ | 0.914 | 0.000 |
|  | *p__Firmicutes; c__Bacilli; o__Bacillales; f__Bacillaceae; g__Bacillus* | Ca^2+^ | 0.811 | 0.001 |
|  | *p__Firmicutes; c__Bacilli; o__Bacillales; f__Bacillaceae; g__Bacillus* | urease | -0.734 | 0.007 |
|  | *p__Firmicutes; c__Bacilli; o__Bacillales; f__Bacillaceae; g__Bacillus* | sucrase | 0.712 | 0.009 |
|  | *p__Firmicutes; c__Bacilli; o__Paenibacillales; f__Paenibacillaceae; g__Paenibacillus* | pH | -0.902 | 0.000 |
|  | *p__Firmicutes; c__Bacilli; o__Paenibacillales; f__Paenibacillaceae; g__Paenibacillus* | EC | 0.898 | 0.000 |
|  | *p__Firmicutes; c__Bacilli; o__Paenibacillales; f__Paenibacillaceae; g__Paenibacillus* | Nmin | 0.832 | 0.001 |
|  | *p__Firmicutes; c__Bacilli; o__Paenibacillales; f__Paenibacillaceae; g__Paenibacillus* | K^+^ | 0.809 | 0.001 |
|  | *p__Firmicutes; c__Bacilli; o__Paenibacillales; f__Paenibacillaceae; g__Paenibacillus* | Ca^2+^ | 0.734 | 0.007 |
|  | *p__Firmicutes; c__Bacilli; o__Paenibacillales; f__Paenibacillaceae; g__Paenibacillus* | urease | -0.804 | 0.002 |
|  | *p__Gemmatimonadota; c__Gemmatimonadetes; o__Gemmatimonadales; f__Gemmatimonadaceae; g__norank_f__Gemmatimonadaceae* | pH | 0.937 | 0.000 |
|  | *p__Gemmatimonadota; c__Gemmatimonadetes; o__Gemmatimonadales; f__Gemmatimonadaceae; g__norank_f__Gemmatimonadaceae* | EC | -0.951 | 0.000 |
|  | *p__Gemmatimonadota; c__Gemmatimonadetes; o__Gemmatimonadales; f__Gemmatimonadaceae; g__norank_f__Gemmatimonadaceae* | Nmin | -0.944 | 0.000 |
|  | *p__Gemmatimonadota; c__Gemmatimonadetes; o__Gemmatimonadales; f__Gemmatimonadaceae; g__norank_f__Gemmatimonadaceae* | K^+^ | -0.767 | 0.004 |
|  | *p__Gemmatimonadota; c__Gemmatimonadetes; o__Gemmatimonadales; f__Gemmatimonadaceae; g__norank_f__Gemmatimonadaceae* | Ca^2+^ | -0.664 | 0.018 |
|  | *p__Gemmatimonadota; c__Gemmatimonadetes; o__Gemmatimonadales; f__Gemmatimonadaceae; g__norank_f__Gemmatimonadaceae* | SOM | 0.650 | 0.022 |
|  | *p__Gemmatimonadota; c__Gemmatimonadetes; o__Gemmatimonadales; f__Gemmatimonadaceae; g__norank_f__Gemmatimonadaceae* | urease | 0.860 | 0.000 |
|  | *p__Patescibacteria; c__Saccharimonadia; o__Saccharimonadales; f__LWQ8; g__norank_f__LWQ8* | pH | -0.722 | 0.008 |
|  | *p__Patescibacteria; c__Saccharimonadia; o__Saccharimonadales; f__LWQ8; g__norank_f__LWQ8* | Nmin | 0.620 | 0.032 |
|  | *p__Patescibacteria; c__Saccharimonadia; o__Saccharimonadales; f__LWQ8; g__norank_f__LWQ8* | K^+^ | 0.688 | 0.013 |
|  | *p__Patescibacteria; c__Saccharimonadia; o__Saccharimonadales; f__LWQ8; g__norank_f__LWQ8* | Ca^2+^ | 0.581 | 0.047 |
|  | *p__Patescibacteria; c__Saccharimonadia; o__Saccharimonadales; f__LWQ8; g__norank_f__LWQ8* | urease | -0.623 | 0.030 |
|  | *p__Planctomycetota; c__Planctomycetes; o__Gemmatales; f__Gemmataceae; g__norank_f__Gemmataceae* | Ca^2+^ | 0.636 | 0.026 |
|  | *p__Proteobacteria; c__Alphaproteobacteria; o__Acetobacterales; f__Acetobacteraceae; g__unclassified_f__Acetobacteraceae* | pH | -0.888 | 0.000 |
|  | *p__Proteobacteria; c__Alphaproteobacteria; o__Acetobacterales; f__Acetobacteraceae; g__unclassified_f__Acetobacteraceae* | EC | 0.908 | 0.000 |
|  | *p__Proteobacteria; c__Alphaproteobacteria; o__Acetobacterales; f__Acetobacteraceae; g__unclassified_f__Acetobacteraceae* | Nmin | 0.888 | 0.000 |
|  | *p__Proteobacteria; c__Alphaproteobacteria; o__Acetobacterales; f__Acetobacteraceae; g__unclassified_f__Acetobacteraceae* | K^+^ | 0.658 | 0.020 |
|  | *p__Proteobacteria; c__Alphaproteobacteria; o__Acetobacterales; f__Acetobacteraceae; g__unclassified_f__Acetobacteraceae* | SOM | -0.762 | 0.004 |
|  | *p__Proteobacteria; c__Alphaproteobacteria; o__Acetobacterales; f__Acetobacteraceae; g__unclassified_f__Acetobacteraceae* | urease | -0.839 | 0.001 |
|  | *p__Proteobacteria; c__Alphaproteobacteria; o__Elsterales; f__norank_o__Elsterales; g__norank_f__norank_o__Elsterales* | pH | 0.671 | 0.017 |
|  | *p__Proteobacteria; c__Alphaproteobacteria; o__Elsterales; f__norank_o__Elsterales; g__norank_f__norank_o__Elsterales* | EC | -0.613 | 0.034 |
|  | *p__Proteobacteria; c__Alphaproteobacteria; o__Elsterales; f__norank_o__Elsterales; g__norank_f__norank_o__Elsterales* | Nmin | -0.657 | 0.020 |
|  | *p__Proteobacteria; c__Alphaproteobacteria; o__Elsterales; f__norank_o__Elsterales; g__norank_f__norank_o__Elsterales* | K^+^ | -0.760 | 0.004 |
|  | *p__Proteobacteria; c__Alphaproteobacteria; o__Elsterales; f__norank_o__Elsterales; g__norank_f__norank_o__Elsterales* | Ca^2+^ | -0.783 | 0.003 |
|  | *p__Proteobacteria; c__Alphaproteobacteria; o__Elsterales; f__norank_o__Elsterales; g__norank_f__norank_o__Elsterales* | urease | 0.580 | 0.048 |
|  | *p__Proteobacteria; c__Alphaproteobacteria; o__Elsterales; f__norank_o__Elsterales; g__norank_f__norank_o__Elsterales* | sucrase | -0.674 | 0.016 |
|  | *p__Proteobacteria; c__Alphaproteobacteria; o__Rhizobiales; f__Beijerinckiaceae; g__Roseiarcus* | pH | 0.860 | 0.000 |
|  | *p__Proteobacteria; c__Alphaproteobacteria; o__Rhizobiales; f__Beijerinckiaceae; g__Roseiarcus* | EC | -0.782 | 0.003 |
|  | *p__Proteobacteria; c__Alphaproteobacteria; o__Rhizobiales; f__Beijerinckiaceae; g__Roseiarcus* | Nmin | -0.825 | 0.001 |
|  | *p__Proteobacteria; c__Alphaproteobacteria; o__Rhizobiales; f__Beijerinckiaceae; g__Roseiarcus* | K^+^ | -0.637 | 0.026 |
|  | *p__Proteobacteria; c__Alphaproteobacteria; o__Rhizobiales; f__Beijerinckiaceae; g__Roseiarcus* | urease | 0.839 | 0.001 |
|  | *p__Proteobacteria; c__Alphaproteobacteria; o__Rhizobiales; f__Xanthobacteraceae; g__Bradyrhizobium* | pH | 0.783 | 0.003 |
|  | *p__Proteobacteria; c__Alphaproteobacteria; o__Rhizobiales; f__Xanthobacteraceae; g__Bradyrhizobium* | EC | -0.803 | 0.002 |
|  | *p__Proteobacteria; c__Alphaproteobacteria; o__Rhizobiales; f__Xanthobacteraceae; g__Bradyrhizobium* | Nmin | -0.839 | 0.001 |
|  | *p__Proteobacteria; c__Alphaproteobacteria; o__Rhizobiales; f__Xanthobacteraceae; g__Bradyrhizobium* | SOM | 0.580 | 0.048 |
|  | *p__Proteobacteria; c__Alphaproteobacteria; o__Rhizobiales; f__Xanthobacteraceae; g__Bradyrhizobium* | urease | 0.790 | 0.002 |
|  | *p__Proteobacteria; c__Alphaproteobacteria; o__Rhizobiales; f__Xanthobacteraceae; g__Bradyrhizobium* | acid phosphata-se | 0.720 | 0.008 |
|  | *p__Proteobacteria; c__Alphaproteobacteria; o__Rhizobiales; f__Xanthobacteraceae; g__norank_f__Xanthobacteraceae* | pH | 0.881 | 0.000 |
|  | *p__Proteobacteria; c__Alphaproteobacteria; o__Rhizobiales; f__Xanthobacteraceae; g__norank_f__Xanthobacteraceae* | EC | -0.856 | 0.000 |
|  | *p__Proteobacteria; c__Alphaproteobacteria; o__Rhizobiales; f__Xanthobacteraceae; g__norank_f__Xanthobacteraceae* | Nmin | -0.881 | 0.000 |
|  | *p__Proteobacteria; c__Alphaproteobacteria; o__Rhizobiales; f__Xanthobacteraceae; g__norank_f__Xanthobacteraceae* | K^+^ | -0.718 | 0.009 |
|  | *p__Proteobacteria; c__Alphaproteobacteria; o__Rhizobiales; f__Xanthobacteraceae; g__norank_f__Xanthobacteraceae* | Ca^2+^ | -0.643 | 0.024 |
|  | *p__Proteobacteria; c__Alphaproteobacteria; o__Rhizobiales; f__Xanthobacteraceae; g__norank_f__Xanthobacteraceae* | SOM | 0.594 | 0.042 |
|  | *p__Proteobacteria; c__Alphaproteobacteria; o__Rhizobiales; f__Xanthobacteraceae; g__norank_f__Xanthobacteraceae* | urease | 0.839 | 0.001 |
|  | *p__Proteobacteria; c__Alphaproteobacteria; o__Rhizobiales; f__Xanthobacteraceae; g__unclassified_f__Xanthobacteraceae* | pH | 0.923 | 0.000 |
|  | *p__Proteobacteria; c__Alphaproteobacteria; o__Rhizobiales; f__Xanthobacteraceae; g__unclassified_f__Xanthobacteraceae* | EC | -0.908 | 0.000 |
|  | *p__Proteobacteria; c__Alphaproteobacteria; o__Rhizobiales; f__Xanthobacteraceae; g__unclassified_f__Xanthobacteraceae* | Nmin | -0.909 | 0.000 |
|  | *p__Proteobacteria; c__Alphaproteobacteria; o__Rhizobiales; f__Xanthobacteraceae; g__unclassified_f__Xanthobacteraceae* | K^+^ | -0.778 | 0.003 |
|  | *p__Proteobacteria; c__Alphaproteobacteria; o__Rhizobiales; f__Xanthobacteraceae; g__unclassified_f__Xanthobacteraceae* | Ca^2+^ | -0.678 | 0.015 |
|  | *p__Proteobacteria; c__Alphaproteobacteria; o__Rhizobiales; f__Xanthobacteraceae; g__unclassified_f__Xanthobacteraceae* | SOM | 0.615 | 0.033 |
|  | *p__Proteobacteria; c__Alphaproteobacteria; o__Rhizobiales; f__Xanthobacteraceae; g__unclassified_f__Xanthobacteraceae* | urease | 0.846 | 0.001 |
|  | *p__Proteobacteria; c__Alphaproteobacteria; o__Sphingomonadales; f__Sphingomonadaceae; g__Sphingomonas* | pH | 0.753 | 0.005 |
|  | *p__Proteobacteria; c__Alphaproteobacteria; o__Sphingomonadales; f__Sphingomonadaceae; g__Sphingomonas* | EC | -0.757 | 0.004 |
|  | *p__Proteobacteria; c__Alphaproteobacteria; o__Sphingomonadales; f__Sphingomonadaceae; g__Sphingomonas* | Nmin | -0.750 | 0.005 |
|  | *p__Proteobacteria; c__Alphaproteobacteria; o__Sphingomonadales; f__Sphingomonadaceae; g__Sphingomonas* | urease | 0.680 | 0.015 |
|  | *p__Proteobacteria; c__Alphaproteobacteria; o__Sphingomonadales; f__Sphingomonadaceae; g__Sphingomonas* | acid phosphata-se | 0.799 | 0.002 |
|  | *p__Proteobacteria; c__Gammaproteobacteria; o__KF-JG30-C25; f__norank_o__KF-JG30-C25; g__norank_f__norank_o__KF-JG30-C25* | acid phosphata-se | -0.720 | 0.008 |
|  | *p__Proteobacteria; c__Gammaproteobacteria; o__Xanthomonadales; f__Rhodanobacteraceae; g__Chujaibacter* | pH | -0.972 | 0.000 |
|  | *p__Proteobacteria; c__Gammaproteobacteria; o__Xanthomonadales; f__Rhodanobacteraceae; g__Chujaibacter* | EC | 0.961 | 0.000 |
|  | *p__Proteobacteria; c__Gammaproteobacteria; o__Xanthomonadales; f__Rhodanobacteraceae; g__Chujaibacter* | Nmin | 0.923 | 0.000 |
|  | *p__Proteobacteria; c__Gammaproteobacteria; o__Xanthomonadales; f__Rhodanobacteraceae; g__Chujaibacter* | K^+^ | 0.750 | 0.005 |
|  | *p__Proteobacteria; c__Gammaproteobacteria; o__Xanthomonadales; f__Rhodanobacteraceae; g__Chujaibacter* | Ca^2+^ | 0.636 | 0.026 |
|  | *p__Proteobacteria; c__Gammaproteobacteria; o__Xanthomonadales; f__Rhodanobacteraceae; g__Chujaibacter* | SOM | -0.622 | 0.031 |
|  | *p__Proteobacteria; c__Gammaproteobacteria; o__Xanthomonadales; f__Rhodanobacteraceae; g__Chujaibacter* | urease | -0.902 | 0.000 |
|  | *p__WPS-2; c__norank_p__WPS-2; o__norank_c__norank_p__WPS-2; f__norank_o__norank_c__norank_p__WPS-2; g__norank_f__norank_o__norank_c__norank_p__WPS-2* | pH | 0.776 | 0.003 |
|  | *p__WPS-2; c__norank_p__WPS-2; o__norank_c__norank_p__WPS-2; f__norank_o__norank_c__norank_p__WPS-2; g__norank_f__norank_o__norank_c__norank_p__WPS-2* | EC | -0.866 | 0.000 |
|  | *p__WPS-2; c__norank_p__WPS-2; o__norank_c__norank_p__WPS-2; f__norank_o__norank_c__norank_p__WPS-2; g__norank_f__norank_o__norank_c__norank_p__WPS-2* | Nmin | -0.790 | 0.002 |
|  | *p__WPS-2; c__norank_p__WPS-2; o__norank_c__norank_p__WPS-2; f__norank_o__norank_c__norank_p__WPS-2; g__norank_f__norank_o__norank_c__norank_p__WPS-2* | K^+^ | -0.641 | 0.025 |
|  | *p__WPS-2; c__norank_p__WPS-2; o__norank_c__norank_p__WPS-2; f__norank_o__norank_c__norank_p__WPS-2; g__norank_f__norank_o__norank_c__norank_p__WPS-2* | SOM | 0.629 | 0.028 |
|  | *p__WPS-2; c__norank_p__WPS-2; o__norank_c__norank_p__WPS-2; f__norank_o__norank_c__norank_p__WPS-2; g__norank_f__norank_o__norank_c__norank_p__WPS-2* | urease | 0.692 | 0.013 |
| Fungal | *p__Ascomycota; c__Dothideomycetes; o__Pleosporales; f__Pleosporaceae; g__Curvularia* | AP | 0.669 | 0.017 |
|  | *p__Ascomycota; c__Dothideomycetes; o__Pleosporales; f__Pleosporaceae; g__Curvularia* | Mg^2+^ | -0.676 | 0.016 |
|  | *p__Ascomycota; c__Eurotiomycetes; o__Chaetothyriales; f__Herpotrichiellaceae; g__Cladophialophora* | pH | -0.608 | 0.036 |
|  | *p__Ascomycota; c__Eurotiomycetes; o__Chaetothyriales; f__Herpotrichiellaceae; g__Cladophialophora* | urease | -0.615 | 0.033 |
|  | *p__Ascomycota; c__Eurotiomycetes; o__Chaetothyriales; f__Herpotrichiellaceae; g__Coniosporium* | Mg^2+^ | -0.741 | 0.006 |
|  | *p__Ascomycota; c__Eurotiomycetes; o__Chaetothyriales; f__Herpotrichiellaceae; g__Coniosporium* | sucrase | -0.691 | 0.013 |
|  | *p__Ascomycota; c__Eurotiomycetes; o__Chaetothyriales; f__unclassified_o__Chaetothyriales; g__unclassified_o__Chaetothyriales* | pH | -0.916 | 0.000 |
|  | *p__Ascomycota; c__Eurotiomycetes; o__Chaetothyriales; f__unclassified_o__Chaetothyriales; g__unclassified_o__Chaetothyriales* | EC | 0.856 | 0.000 |
|  | *p__Ascomycota; c__Eurotiomycetes; o__Chaetothyriales; f__unclassified_o__Chaetothyriales; g__unclassified_o__Chaetothyriales* | Nmin | 0.895 | 0.000 |
|  | *p__Ascomycota; c__Eurotiomycetes; o__Chaetothyriales; f__unclassified_o__Chaetothyriales; g__unclassified_o__Chaetothyriales* | K^+^ | 0.623 | 0.030 |
|  | *p__Ascomycota; c__Eurotiomycetes; o__Chaetothyriales; f__unclassified_o__Chaetothyriales; g__unclassified_o__Chaetothyriales* | SOM | -0.594 | 0.042 |
|  | *p__Ascomycota; c__Eurotiomycetes; o__Chaetothyriales; f__unclassified_o__Chaetothyriales; g__unclassified_o__Chaetothyriales* | urease | -0.944 | 0.000 |
|  | *p__Ascomycota; c__Eurotiomycetes; o__Eurotiales; f__Aspergillaceae; g__Aspergillus* | Mg^2+^ | 0.692 | 0.013 |
|  | *p__Ascomycota; c__Eurotiomycetes; o__Eurotiales; f__Aspergillaceae; g__Aspergillus* | sucrase | 0.600 | 0.039 |
|  | *p__Ascomycota; c__Eurotiomycetes; o__Eurotiales; f__Aspergillaceae; g__Penicillium* | Mg^2+^ | -0.720 | 0.008 |
|  | *p__Ascomycota; c__Eurotiomycetes; o__Eurotiales; f__Aspergillaceae; g__unclassified_f__Aspergillaceae* | K^+^ | -0.621 | 0.031 |
|  | *p__Ascomycota; c__Eurotiomycetes; o__Eurotiales; f__Aspergillaceae; g__unclassified_f__Aspergillaceae* | Ca^2+^ | -0.711 | 0.010 |
|  | *p__Ascomycota; c__Eurotiomycetes; o__Eurotiales; f__Aspergillaceae; g__unclassified_f__Aspergillaceae* | Mg^2+^ | -0.666 | 0.018 |
|  | *p__Ascomycota; c__Eurotiomycetes; o__Eurotiales; f__Aspergillaceae; g__unclassified_f__Aspergillaceae* | sucrase | -0.921 | 0.000 |
|  | *p__Ascomycota; c__Eurotiomycetes; o__Eurotiales; f__Trichocomaceae; g__Sagenomella* | AP | 0.657 | 0.020 |
|  | *p__Ascomycota; c__Eurotiomycetes; o__Eurotiales; f__Trichocomaceae; g__Sagenomella* | Mg^2+^ | -0.853 | 0.000 |
|  | *p__Ascomycota; c__Eurotiomycetes; o__Eurotiales; f__Trichocomaceae; g__Talaromyces* | pH | -0.657 | 0.020 |
|  | *p__Ascomycota; c__Eurotiomycetes; o__Eurotiales; f__Trichocomaceae; g__Talaromyces* | EC | 0.623 | 0.030 |
|  | *p__Ascomycota; c__Eurotiomycetes; o__Eurotiales; f__Trichocomaceae; g__Talaromyces* | Nmin | 0.615 | 0.033 |
|  | *p__Ascomycota; c__Eurotiomycetes; o__Eurotiales; f__Trichocomaceae; g__Talaromyces* | urease | -0.615 | 0.033 |
|  | *p__Ascomycota; c__Eurotiomycetes; o__Eurotiales; f__Trichocomaceae; g__Talaromyces* | acid phosphata-se | -0.741 | 0.006 |
|  | *p__Ascomycota; c__Eurotiomycetes; o__Eurotiales; f__unclassified_o__Eurotiales; g__unclassified_o__Eurotiales* | K^+^ | -0.635 | 0.026 |
|  | *p__Ascomycota; c__Leotiomycetes; o__Helotiales; f__Helotiales_fam_Incertae_sedis; g__Kendrickiella* | Ca^2+^ | 0.592 | 0.043 |
|  | *p__Ascomycota; c__Leotiomycetes; o__Helotiales; f__Helotiales_fam_Incertae_sedis; g__Kendrickiella* | Mg^2+^ | 0.746 | 0.005 |
|  | *p__Ascomycota; c__Leotiomycetes; o__Helotiales; f__Helotiales_fam_Incertae_sedis; g__Kendrickiella* | sucrase | 0.770 | 0.003 |
|  | *p__Ascomycota; c__Leotiomycetes; o__Helotiales; f__Vibrisseaceae; g__Phialocephala* | pH | 0.662 | 0.019 |
|  | *p__Ascomycota; c__Leotiomycetes; o__Helotiales; f__Vibrisseaceae; g__Phialocephala* | EC | -0.635 | 0.027 |
|  | *p__Ascomycota; c__Leotiomycetes; o__Helotiales; f__Vibrisseaceae; g__Phialocephala* | Nmin | -0.708 | 0.010 |
|  | *p__Ascomycota; c__Leotiomycetes; o__Helotiales; f__Vibrisseaceae; g__Phialocephala* | SOM | 0.725 | 0.008 |
|  | *p__Ascomycota; c__Leotiomycetes; o__Helotiales; f__Vibrisseaceae; g__Phialocephala* | urease | 0.669 | 0.017 |
|  | *p__Ascomycota; c__Sordariomycetes; o__Chaetosphaeriales; f__Chaetosphaeriaceae; g__Chaetosphaeria* | pH | -0.580 | 0.048 |
|  | *p__Ascomycota; c__Sordariomycetes; o__Chaetosphaeriales; f__Chaetosphaeriaceae; g__Chaetosphaeria* | EC | 0.602 | 0.038 |
|  | *p__Ascomycota; c__Sordariomycetes; o__Chaetosphaeriales; f__Chaetosphaeriaceae; g__Gonytrichum* | AP | -0.592 | 0.043 |
|  | *p__Ascomycota; c__Sordariomycetes; o__Chaetosphaeriales; f__Chaetosphaeriaceae; g__Gonytrichum* | SOM | 0.865 | 0.000 |
|  | *p__Ascomycota; c__Sordariomycetes; o__Coniochaetales; f__unclassified_o__Coniochaetales; g__unclassified_o__Coniochaetales* | AP | -0.807 | 0.002 |
|  | *p__Ascomycota; c__Sordariomycetes; o__Coniochaetales; f__unclassified_o__Coniochaetales; g__unclassified_o__Coniochaetales* | K^+^ | -0.600 | 0.039 |
|  | *p__Ascomycota; c__Sordariomycetes; o__Coniochaetales; f__unclassified_o__Coniochaetales; g__unclassified_o__Coniochaetales* | SOM | 0.691 | 0.013 |
|  | *p__Ascomycota; c__Sordariomycetes; o__Diaporthales; f__Melanconidaceae; g__Melanconiella* | pH | 0.944 | 0.000 |
|  | *p__Ascomycota; c__Sordariomycetes; o__Diaporthales; f__Melanconidaceae; g__Melanconiella* | EC | -0.982 | 0.000 |
|  | *p__Ascomycota; c__Sordariomycetes; o__Diaporthales; f__Melanconidaceae; g__Melanconiella* | Nmin | -0.958 | 0.000 |
|  | *p__Ascomycota; c__Sordariomycetes; o__Diaporthales; f__Melanconidaceae; g__Melanconiella* | K^+^ | -0.746 | 0.005 |
|  | *p__Ascomycota; c__Sordariomycetes; o__Diaporthales; f__Melanconidaceae; g__Melanconiella* | Ca^2+^ | -0.671 | 0.017 |
|  | *p__Ascomycota; c__Sordariomycetes; o__Diaporthales; f__Melanconidaceae; g__Melanconiella* | SOM | 0.713 | 0.009 |
|  | *p__Ascomycota; c__Sordariomycetes; o__Diaporthales; f__Melanconidaceae; g__Melanconiella* | urease | 0.867 | 0.000 |
|  | *p__Ascomycota; c__Sordariomycetes; o__Hypocreales; f__Clavicipitaceae; g__Collarina* | AP | -0.687 | 0.014 |
|  | *p__Ascomycota; c__Sordariomycetes; o__Hypocreales; f__Clavicipitaceae; g__Collarina* | Mg^2+^ | 0.669 | 0.017 |
|  | *p__Ascomycota; c__Sordariomycetes; o__Hypocreales; f__Clavicipitaceae; g__Collarina* | SOM | 0.616 | 0.033 |
|  | *p__Ascomycota; c__Sordariomycetes; o__Hypocreales; f__Hypocreaceae; g__Trichoderma* | AP | 0.678 | 0.015 |
|  | *p__Ascomycota; c__Sordariomycetes; o__Hypocreales; f__Hypocreales_fam_Incertae_sedis; g__Acremonium* | AP | 0.783 | 0.003 |
|  | *p__Ascomycota; c__Sordariomycetes; o__Hypocreales; f__Hypocreales_fam_Incertae_sedis; g__Acremonium* | Mg^2+^ | -0.699 | 0.011 |
|  | *p__Ascomycota; c__Sordariomycetes; o__Hypocreales; f__Nectriaceae; g__Fusarium* | pH | -0.594 | 0.042 |
|  | *p__Ascomycota; c__Sordariomycetes; o__Hypocreales; f__Nectriaceae; g__Fusarium* | EC | 0.655 | 0.021 |
|  | *p__Ascomycota; c__Sordariomycetes; o__Hypocreales; f__Nectriaceae; g__Fusarium* | Nmin | 0.629 | 0.028 |
|  | *p__Ascomycota; c__Sordariomycetes; o__Hypocreales; f__Nectriaceae; g__Fusarium* | AP | 0.650 | 0.022 |
|  | *p__Ascomycota; c__Sordariomycetes; o__Hypocreales; f__Nectriaceae; g__Fusarium* | K^+^ | 0.683 | 0.014 |
|  | *p__Ascomycota; c__Sordariomycetes; o__Hypocreales; f__Nectriaceae; g__Fusarium* | Ca^2+^ | 0.692 | 0.013 |
|  | *p__Ascomycota; c__Sordariomycetes; o__Hypocreales; f__Nectriaceae; g__Fusarium* | SOM | -0.587 | 0.045 |
|  | *p__Ascomycota; c__Sordariomycetes; o__Hypocreales; f__Ophiocordycipitaceae; g__Purpureocillium* | pH | 0.606 | 0.037 |
|  | *p__Ascomycota; c__Sordariomycetes; o__Hypocreales; f__Ophiocordycipitaceae; g__Purpureocillium* | Nmin | -0.620 | 0.032 |
|  | *p__Ascomycota; c__Sordariomycetes; o__Hypocreales; f__Ophiocordycipitaceae; g__Purpureocillium* | K^+^ | -0.718 | 0.009 |
|  | *p__Ascomycota; c__Sordariomycetes; o__Hypocreales; f__Ophiocordycipitaceae; g__Purpureocillium* | Ca^2+^ | -0.585 | 0.046 |
|  | *p__Ascomycota; c__Sordariomycetes; o__Hypocreales; f__Stachybotryaceae; g__unclassified_f__Stachybotryaceae* | AP | 0.613 | 0.034 |
|  | *p__Ascomycota; c__Sordariomycetes; o__Hypocreales; f__unclassified_o__Hypocreales; g__unclassified_o__Hypocreales* | AP | -0.629 | 0.028 |
|  | *p__Ascomycota; c__Sordariomycetes; o__Hypocreales; f__unclassified_o__Hypocreales; g__unclassified_o__Hypocreales* | Mg^2+^ | 0.699 | 0.011 |
|  | *p__Ascomycota; c__Sordariomycetes; o__Hypocreales; f__unclassified_o__Hypocreales; g__unclassified_o__Hypocreales* | SOM | 0.692 | 0.013 |
|  | *p__Ascomycota; c__Sordariomycetes; o__Microascales; f__Microascaceae; g__Cephalotrichum* | Mg^2+^ | -0.697 | 0.012 |
|  | *p__Ascomycota; c__Sordariomycetes; o__Microascales; f__Microascaceae; g__Cephalotrichum* | sucrase | -0.775 | 0.003 |
|  | *p__Ascomycota; c__Sordariomycetes; o__Ophiostomatales; f__Ophiostomataceae; g__Hawksworthiomyces* | Mg^2+^ | -0.939 | 0.000 |
|  | *p__Ascomycota; c__Sordariomycetes; o__Ophiostomatales; f__Ophiostomataceae; g__Sporothrix* | AP | 0.944 | 0.000 |
|  | *p__Ascomycota; c__Sordariomycetes; o__Ophiostomatales; f__Ophiostomataceae; g__Sporothrix* | K^+^ | 0.606 | 0.037 |
|  | *p__Ascomycota; c__Sordariomycetes; o__Sordariales; f__Chaetomiaceae; g__Arcopilus* | Mg^2+^ | -0.615 | 0.033 |
|  | *p__Ascomycota; c__Sordariomycetes; o__Sordariales; f__Chaetomiaceae; g__Myceliophthora* | SOM | -0.594 | 0.042 |
|  | *p__Ascomycota; c__Sordariomycetes; o__Trichosphaeriales; f__Trichosphaeriaceae; g__Nigrospora* | Mg^2+^ | -0.837 | 0.001 |
|  | *p__Ascomycota; c__Sordariomycetes; o__Trichosphaeriales; f__Trichosphaeriaceae; g__Nigrospora* | sucrase | -0.664 | 0.018 |
|  | *p__Ascomycota; c__Sordariomycetes; o__unclassified_c__Sordariomycetes; f__unclassified_c__Sordariomycetes; g__unclassified_c__Sordariomycetes* | pH | 0.923 | 0.000 |
|  | *p__Ascomycota; c__Sordariomycetes; o__unclassified_c__Sordariomycetes; f__unclassified_c__Sordariomycetes; g__unclassified_c__Sordariomycetes* | EC | -0.898 | 0.000 |
|  | *p__Ascomycota; c__Sordariomycetes; o__unclassified_c__Sordariomycetes; f__unclassified_c__Sordariomycetes; g__unclassified_c__Sordariomycetes* | Nmin | -0.895 | 0.000 |
|  | *p__Ascomycota; c__Sordariomycetes; o__unclassified_c__Sordariomycetes; f__unclassified_c__Sordariomycetes; g__unclassified_c__Sordariomycetes* | K^+^ | -0.666 | 0.018 |
|  | *p__Ascomycota; c__Sordariomycetes; o__unclassified_c__Sordariomycetes; f__unclassified_c__Sordariomycetes; g__unclassified_c__Sordariomycetes* | urease | 0.874 | 0.000 |
|  | *p__Ascomycota; c__unclassified_p__Ascomycota; o__unclassified_p__Ascomycota; f__unclassified_p__Ascomycota; g__unclassified_p__Ascomycota* | pH | -0.678 | 0.015 |
|  | *p__Ascomycota; c__unclassified_p__Ascomycota; o__unclassified_p__Ascomycota; f__unclassified_p__Ascomycota; g__unclassified_p__Ascomycota* | EC | 0.676 | 0.016 |
|  | *p__Ascomycota; c__unclassified_p__Ascomycota; o__unclassified_p__Ascomycota; f__unclassified_p__Ascomycota; g__unclassified_p__Ascomycota* | Nmin | 0.692 | 0.013 |
|  | *p__Ascomycota; c__unclassified_p__Ascomycota; o__unclassified_p__Ascomycota; f__unclassified_p__Ascomycota; g__unclassified_p__Ascomycota* | SOM | -0.678 | 0.015 |
|  | *p__Ascomycota; c__unclassified_p__Ascomycota; o__unclassified_p__Ascomycota; f__unclassified_p__Ascomycota; g__unclassified_p__Ascomycota* | urease | -0.811 | 0.001 |
|  | *p__Ascomycota; c__unclassified_p__Ascomycota; o__unclassified_p__Ascomycota; f__unclassified_p__Ascomycota; g__unclassified_p__Ascomycota* | acid phosphata-se | -0.741 | 0.006 |
|  | *p__Basidiomycota; c__Agaricomycetes; o__Agaricales; f__Agaricaceae; g__unclassified_f__Agaricaceae* | AP | 0.734 | 0.007 |
|  | *p__Basidiomycota; c__Agaricomycetes; o__Agaricales; f__Agaricaceae; g__unclassified_f__Agaricaceae* | acid phosphata-se | 0.725 | 0.008 |
|  | *p__Basidiomycota; c__Agaricomycetes; o__Agaricales; f__unclassified_o__Agaricales; g__unclassified_o__Agaricales* | AP | 0.684 | 0.014 |
|  | *p__Basidiomycota; c__Agaricomycetes; o__Agaricales; f__unclassified_o__Agaricales; g__unclassified_o__Agaricales* | Mg^2+^ | -0.787 | 0.002 |
|  | *p__Basidiomycota; c__Agaricomycetes; o__Phallales; f__Phallaceae; g__Phallus* | AP | 0.592 | 0.043 |
|  | *p__Basidiomycota; c__Agaricomycetes; o__Phallales; f__Phallaceae; g__Phallus* | Mg^2+^ | -0.655 | 0.021 |
|  | *p__Basidiomycota; c__Agaricomycetes; o__Phallales; f__Phallaceae; g__Phallus* | acid phosphata-se | 0.627 | 0.029 |
|  | *p__Basidiomycota; c__Agaricomycetes; o__Trechisporales; f__Hydnodontaceae; g__Trechispora* | pH | -0.762 | 0.004 |
|  | *p__Basidiomycota; c__Agaricomycetes; o__Trechisporales; f__Hydnodontaceae; g__Trechispora* | EC | 0.782 | 0.003 |
|  | *p__Basidiomycota; c__Agaricomycetes; o__Trechisporales; f__Hydnodontaceae; g__Trechispora* | Nmin | 0.755 | 0.005 |
|  | *p__Basidiomycota; c__Agaricomycetes; o__Trechisporales; f__Hydnodontaceae; g__Trechispora* | SOM | -0.685 | 0.014 |
|  | *p__Basidiomycota; c__Agaricomycetes; o__Trechisporales; f__Hydnodontaceae; g__Trechispora* | urease | -0.776 | 0.003 |
|  | *p__Basidiomycota; c__Tremellomycetes; o__Tremellales; f__Rhynchogastremataceae; g__Papiliotrema* | AP | 0.727 | 0.007 |
|  | *p__Basidiomycota; c__Tremellomycetes; o__Tremellales; f__Rhynchogastremataceae; g__Papiliotrema* | Mg^2+^ | -0.594 | 0.042 |
|  | *p__Basidiomycota; c__Tremellomycetes; o__Tremellales; f__unclassified_o__Tremellales; g__unclassified_o__Tremellales* | pH | -0.860 | 0.000 |
|  | *p__Basidiomycota; c__Tremellomycetes; o__Tremellales; f__unclassified_o__Tremellales; g__unclassified_o__Tremellales* | EC | 0.887 | 0.000 |
|  | *p__Basidiomycota; c__Tremellomycetes; o__Tremellales; f__unclassified_o__Tremellales; g__unclassified_o__Tremellales* | Nmin | 0.867 | 0.000 |
|  | *p__Basidiomycota; c__Tremellomycetes; o__Tremellales; f__unclassified_o__Tremellales; g__unclassified_o__Tremellales* | K^+^ | 0.806 | 0.002 |
|  | *p__Basidiomycota; c__Tremellomycetes; o__Tremellales; f__unclassified_o__Tremellales; g__unclassified_o__Tremellales* | Ca^2+^ | 0.671 | 0.017 |
|  | *p__Basidiomycota; c__Tremellomycetes; o__Tremellales; f__unclassified_o__Tremellales; g__unclassified_o__Tremellales* | urease | -0.867 | 0.000 |
|  | *p__Chytridiomycota; c__unclassified_p__Chytridiomycota; o__unclassified_p__Chytridiomycota; f__unclassified_p__Chytridiomycota; g__unclassified_p__Chytridiomycota* | pH | 0.588 | 0.044 |
|  | *p__Chytridiomycota; c__unclassified_p__Chytridiomycota; o__unclassified_p__Chytridiomycota; f__unclassified_p__Chytridiomycota; g__unclassified_p__Chytridiomycota* | EC | -0.646 | 0.023 |
|  | *p__Chytridiomycota; c__unclassified_p__Chytridiomycota; o__unclassified_p__Chytridiomycota; f__unclassified_p__Chytridiomycota; g__unclassified_p__Chytridiomycota* | K^+^ | -0.639 | 0.025 |
|  | *p__Chytridiomycota; c__unclassified_p__Chytridiomycota; o__unclassified_p__Chytridiomycota; f__unclassified_p__Chytridiomycota; g__unclassified_p__Chytridiomycota* | Ca^2+^ | -0.637 | 0.026 |
|  | *p__Mortierellomycota; c__Mortierellomycetes; o__Mortierellales; f__Mortierellaceae; g__Mortierella* | K^+^ | -0.767 | 0.004 |
|  | *p__Mortierellomycota; c__Mortierellomycetes; o__Mortierellales; f__Mortierellaceae; g__Mortierella* | Ca^2+^ | -0.832 | 0.001 |
|  | *p__Mortierellomycota; c__Mortierellomycetes; o__Mortierellales; f__Mortierellaceae; g__Mortierella* | Mg^2+^ | -0.601 | 0.039 |
|  | *p__Mortierellomycota; c__Mortierellomycetes; o__Mortierellales; f__Mortierellaceae; g__Mortierella* | sucrase | -0.961 | 0.000 |
|  | *p__unclassified_k__Fungi; c__unclassified_k__Fungi; o__unclassified_k__Fungi; f__unclassified_k__Fungi; g__unclassified_k__Fungi* | pH | 0.846 | 0.001 |
|  | *p__unclassified_k__Fungi; c__unclassified_k__Fungi; o__unclassified_k__Fungi; f__unclassified_k__Fungi; g__unclassified_k__Fungi* | EC | -0.877 | 0.000 |
|  | *p__unclassified_k__Fungi; c__unclassified_k__Fungi; o__unclassified_k__Fungi; f__unclassified_k__Fungi; g__unclassified_k__Fungi* | Nmin | -0.839 | 0.001 |
|  | *p__unclassified_k__Fungi; c__unclassified_k__Fungi; o__unclassified_k__Fungi; f__unclassified_k__Fungi; g__unclassified_k__Fungi* | K^+^ | -0.599 | 0.040 |
|  | *p__unclassified_k__Fungi; c__unclassified_k__Fungi; o__unclassified_k__Fungi; f__unclassified_k__Fungi; g__unclassified_k__Fungi* | urease | 0.790 | 0.002 |
|  | *p__unclassified_k__Fungi; c__unclassified_k__Fungi; o__unclassified_k__Fungi; f__unclassified_k__Fungi; g__unclassified_k__Fungi* | acid phosphata-se | 0.608 | 0.036 |

Nmin, AP, K^+^, Mg^2+^, SOM and Ca^2+^ stand for mineral nitrogen, available phosphorus, available potassium, exchangeable Mg^2+^, exchangeable Ca^2+^, and soil organic carbon, individually. The absolute values of correlation coefficients are more than 0.5 (*P* < 0.05). Species name include bacteria and fungi, and soil factors include Nmin, AP, K^+^, Mg^2+^, Ca^2+^, SOM, EC, pH, urease, acid phosphatase and sucrase activity.

## Supplementary Figures


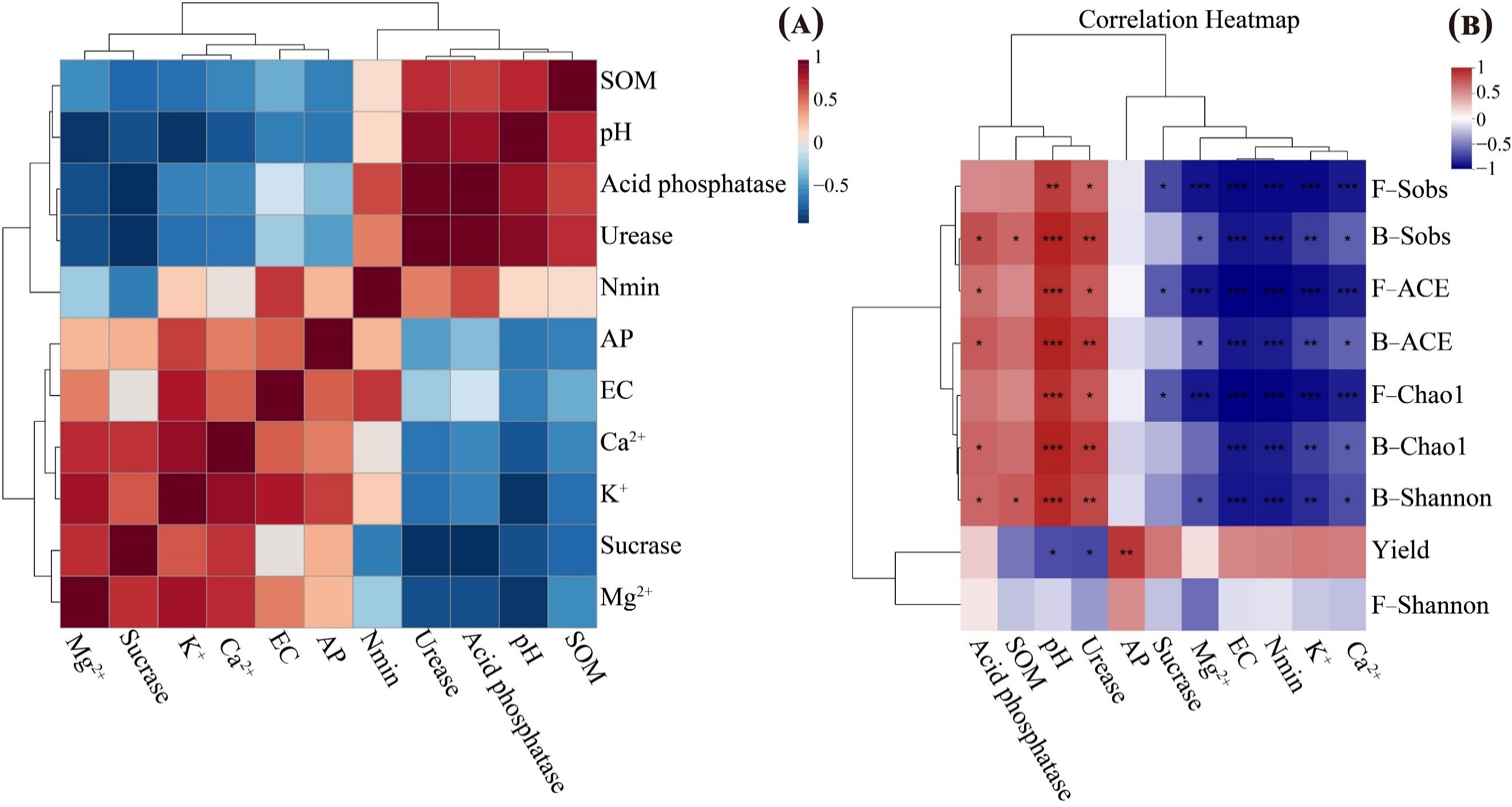


**Supplementary Figure 1.** Correlation matrices between soil agricultural properties and soil enzyme activity (A). Correlation matrices between enzyme activity and yield and microbial diversity indices (Sobs, ACE, Chao1 and Shannon) (B). “*” and “**” mean significant correlation at 0.05 and 0.01 levels, respectively. Nmin, AP, K^+^, Mg^2+^, SOM and Ca^2+^ stand for mineral nitrogen, available phosphorus, available potassium, Exchangeable Mg^2+^, Exchangeable Ca^2+^, and soil organic carbon, individually. B–Sobs, B–ACE, B–Chao1 and B–Shannon stand for bacteria diversity indices. F–Sobs, F–ACE, F–Chao1 and F–Shannon stand for fungal diversity indices.

.


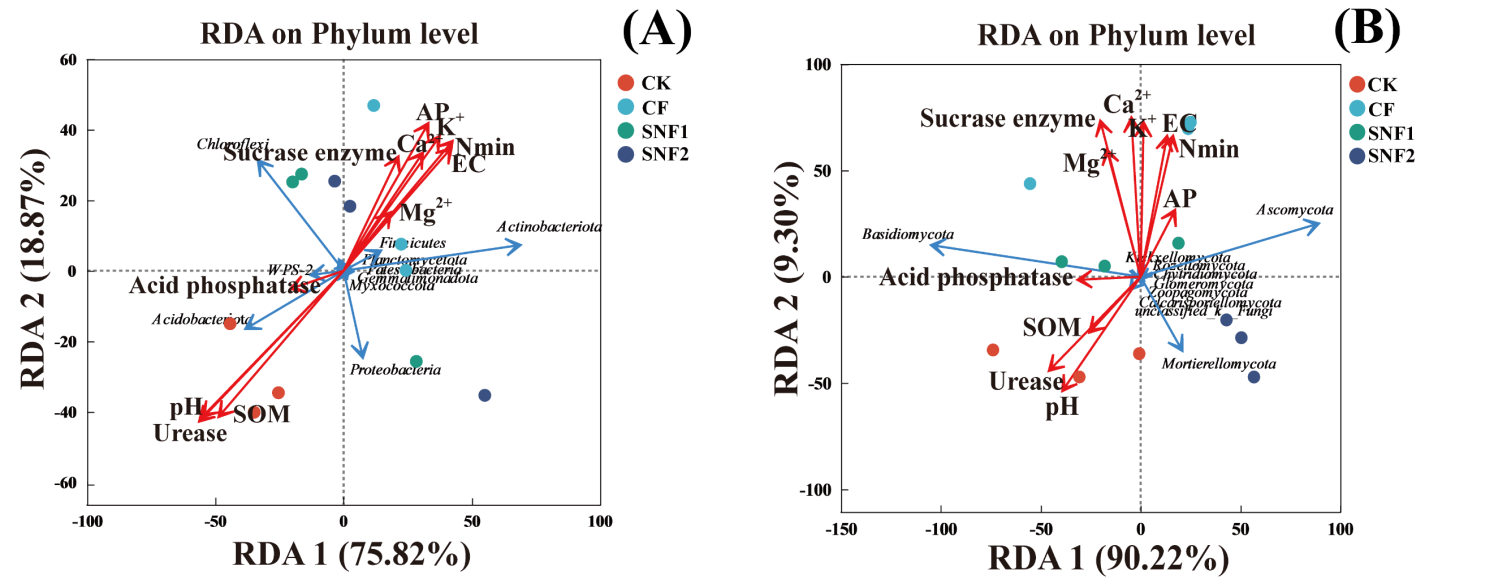


**Supplementary Figure 2.** Redundancy analysis to show the relationships between bacterial (A) and fungal (B) communities and environmental factors in 2020. The dots colored represent different treatments. The red arrows represent environmental factors. The blue arrows represent microbial species. Arrows designate the direction and magnitude of environmental determinants correlated with soil microbial communities. The angles between the red and blue arrow represent their correlations, and the acute and obtuse angles represent positive and negative correlations, respectively. Nmin, AP, K^+^, Mg^2+^, SOM and Ca^2+^ stand for mineral nitrogen, available phosphorus, available potassium, Exchangeable Mg^2+^, Exchangeable Ca^2+^, and soil organic carbon, individually. CK, CF, SNF1 and SNF2 is non fertilizer, conventional fertilizer, 15% less than CF and 20% less than CF, respectively.
